# Circ-GALNT16 restrains colorectal cancer progression by enhancing the SUMOylation of hnRNPK

**DOI:** 10.1186/s13046-021-02074-7

**Published:** 2021-08-27

**Authors:** Chaofan Peng, Yuqian Tan, Peng Yang, Kangpeng Jin, Chuan Zhang, Wen Peng, Lu Wang, Jiahui Zhou, Ranran Chen, Tuo Wang, Chi Jin, Jiangzhou Ji, Yifei Feng, Junwei Tang, Yueming Sun

**Affiliations:** 1 Department of General Surgery, The First Affiliated Hospital of Nanjing Medical University, Jiangsu 210029 Nanjing, People’s Republic of China; 2 The First School of Clinical Medicine, Nanjing Medical University, Nanjing, China; 3 Nanjing Medical University, Nanjing, China

**Keywords:** Colorectal cancer, circ-GALNT16, hnRNPK, SUMOylation, p53, Serpine1, SENP2

## Abstract

**Background:**

Recent studies have investigated the role of circular RNAs (circRNAs) as significant regulatory factors in multiple cancer progression. Nevertheless, the biological functions of circRNAs and the underlying mechanisms by which they regulate colorectal cancer (CRC) progression remain unclear.

**Methods:**

A novel circRNA (circ-GALNT16) was identified by microarray and qRT-PCR. A series of *in vitro* and *in vivo* phenotype experiments were performed to investigate the role of circ-GALNT16 in CRC. The FISH, RNA pulldown assay, RIP assay, RNA sequencing, coimmunoprecipitation, and ChIP were performed to investigate the molecular mechanisms of circ-GALNT16 in CRC progression.

**Results:**

Circ-GALNT16 was downregulated in CRC and was negatively correlated with poor prognosis. Circ-GALNT16 suppressed the proliferation and metastatic ability of CRC cells *in vitro* and *in vivo*. Mechanistically, circ-GALNT16 could bind to the KH3 domain of heterogeneous nuclear ribonucleoprotein K (hnRNPK), which promoted the SUMOylation of hnRNPK. Additionally, circ-GALNT16 could enhance the formation of the hnRNPK-p53 complex by facilitating the SUMOylation of hnRNPK. RNA sequencing assay identified serpin family E member 1 as the target gene of circ-GALNT16 at the transcriptional level. Rescue assays revealed that circ-GALNT16 regulated the expression of Serpine1 by inhibiting the deSUMOylation of hnRNPK mediated by SUMO-specific peptidase 2 and then regulating the sequence-specific DNA binding ability of the hnRNPK-p53 transcriptional complex.

**Conclusions:**

Circ-GALNT16 suppressed CRC progression by inhibiting Serpine1 expression through regulating the sequence-specific DNA binding ability of the SENP2-mediated hnRNPK-p53 transcriptional complex and might function as a biomarker and therapeutic target for CRC.

**Supplementary Information:**

The online version contains supplementary material available at 10.1186/s13046-021-02074-7.

## Background

Colorectal cancer (CRC) is one of the most common malignant tumors and continues to remain a public health concern [1]. The incidence and mortality rate of CRC rank third and second, respectively, among various cancers according to the up-to-date global cancer statistics. The prognoses of patients with advanced CRC remain poor, although neoadjuvant chemoradiotherapy, surgery, postoperative chemoradiotherapy, and immunotherapy are widely used to treat patients with CRC and have been gaining recognition rapidly [2]. One of the main causes for poor prognosis is that the detailed mechanisms of CRC progression remain unclear. Therefore, it is crucial to determine the unknown pathogenic mechanisms of CRC progression to develop specific diagnostic markers and accurate therapeutic targets.

Circular RNA (circRNA) is a kind of transcript that originates from the back-splicing of pre-mRNA [3]. CircRNAs have higher stability than linear transcripts and are more resistant to RNase R because of their loop structure, which makes circRNAs potential diagnostic markers and therapeutic targets for cancers [4, 5]. The biological functions of circRNAs are widely involved in the proliferation, metastasis, apoptosis, and autophagy of various cancers through multiple pathways [6–9]. For example, hsa_circ_101555 can promote CRC progression by acting as a competing endogenous RNA of miR-597-5p [10]. Circ-ABCC1 binds to β-catenin and activates the Wnt/β-catenin pathway to facilitate CRC progression [11]. CircPPP1R12A encodes a novel protein that promotes colon cancer pathogenesis through Hippo-YAP signaling [12]. However, the detailed mechanisms by which circRNAs regulate the progression of CRC remain elusive.

Heterogeneous nuclear ribonucleoprotein K (hnRNPK) is a DNA- and RNA-binding protein that regulates several biological processes, including transcriptional regulation, nucleocytoplasmic transport, and DNA damage repair [13]. Some studies on cancer have identified hnRNPK as an oncogene because of its overexpression in several cancer tissues, while hnRNPK has also been identified as an antioncogene as it activates the p53 pathway. SUMOylation is a type of posttranslational modification of hnRNPK by conjugating a small ubiquitin-like modifier (SUMO) covalently through a multistep reaction [14]. The SUMOylation of hnRNPK may regulate RNA metabolism at the transcriptional and posttranscriptional levels [15]. Several studies have reported that the SUMOylation of hnRNPK could enhance the interaction between hnRNPK and p53 and regulate the transcriptional activity of p53 [16–18].

In the present study, we elucidated whether circ-GALNT16 could suppress CRC progression by enhancing the SUMOylation of hnRNPK to regulate the sequence-specific DNA binding ability of the hnRNPK-p53 complex. Collectively, our study investigated circ-GALNT16-mediated specific molecular mechanisms in CRC progression, which might contribute to determining the suitability of circ-GALNT16 as a potential diagnostic marker and therapeutic target for CRC.

## Methods

### Microarray analysis of circRNAs

The circRNA microarray analysis (CapitalBio Technology, Beijing, China) was performed using specific probes targeting the back-splicing sites of human circRNAs. The circRNAs were detected in five pairs of CRC tissues. Data normalization, summarization, and quality control were performed by the GeneSpring software. The differential circRNAs are listed in Table S1. All the raw data of the microarray analysis are available in the Gene Expression Omnibus (accession number: GSE172229).

### Patient samples and cell culture

A total of 100 tumor samples from patients with CRC and paired adjacent normal tissues were collected between 2014 and 2018 from the Department of General Surgery, First Affiliated Hospital of Nanjing Medical University, Nanjing, China. Fresh tissues were obtained after surgery and immediately frozen at -80 °C. Two ml RNAlater (AM7020, Thermo Fisher Scientific, Waltham, USA) was used to protect the tissue RNA from degradation. Patients who had received neoadjuvant chemoradiotherapy were excluded. This study was approved by the Human Ethics Committee of First Affiliated Hospital of Nanjing Medical University. Each patient was informed about the study and consented to specimen donation before the surgery. The raw data of circ-GALNT16 expression in the 100 pairs of CRC tissues and relative expression in different pathological subgroups are provided in Table S2.

The cell lines LoVo, DLD-1, SW-480, Caco-2, RKO, HCT 116, HT-29, and NCM460 were purchased from the Cell Bank of Type Culture Collection of the Chinese Academy of Sciences (Shanghai, China) and cultured in a recommended medium with 10 % fetal bovine serum at 37 °C in a 5 % CO_2_ humidified incubator.

### RNA extraction and qRT-PCR

Total RNA was extracted from patients’ specimens and cells by TRIzol reagent (Invitrogen, USA), and the extraction procedure was performed as described previously [19]. Then, a PrimeScript RT reagent kit (TaKara, Dalian, China) was used for reverse transcription. An SYBR Premix Ex Taq Kit (TaKara) was used for qRT-PCR on an Applied Biosystems 7500 Sequence Detection System. GAPDH was used as an internal control. The sequences of primers are listed in Table S3.

### Cell transfection

A lentivirus containing short hairpin RNAs (shRNAs) and full-length targeting sequence of circ-GALNT16, including the corresponding negative control, were synthesized by Obio (Shanghai, China). The Serpine1 overexpression plasmid was obtained from Obio. The truncated hnRNPK plasmids of hnRNPK with a C-terminus 3× Flag tag were synthesized by Obio. The small interfering RNA (siRNA) oligonucleotides targeting hnRNPK, SENP2, and Serpine1 were synthesized by RiboBio (Guangzhou, China). The transfection procedures were performed as described previously by using Lipofectamine 3000 (Invitrogen) [20]. The sequences of shRNAs and siRNAs are listed in Table S3.

### Cell proliferation assays

Cell proliferation assays, including Cell Counting Kit-8 assay and colony formation assay, were performed as described previously [21]. The cell proliferation ability was measured by the 5-ethynyl-2ʹ-deoxyuridine assay using an EdU kit (Beyotime, Shanghai, China).

### Transwell and scratch wound healing assay

Transwell assay was performed with Millipore cell culture inserts (24-well insert, 8 μm pore size). For migration assay, 3 × 10^4^ cells resuspended in 200 µL serum-free medium per well were seeded onto the upper chamber of the Transwell membrane, while the lower chamber was filled with 700 µL culture medium containing 10 % serum. After 24 h (36 or 48 h based on different cell lines), we stained the cells adhering to the underside of the membrane by using a Crystal Violet solution and removed the cells above the membrane with swabs. Cells in each well were counted by five random views under a microscope. For invasion assay, Matrigel (BD Biosciences, Franklin Lakes, NJ, USA) was spread on the upper layer, and the remaining steps were performed as described above.

A 200-µL pipette tip was used to produce consistent length lesions in 6-well plates (8 × 10^5^ cells per well) for wound healing assay. An inversion microscope was used to acquire each wound image at 0 and 48 h. We used ImageJ software to quantitatively evaluate the gap distance.

### Flow cytometry assay of cell cycle and apoptosis

Treated cells were fixed in 75 % alcohol overnight at -20 °C for cell cycle assay. The cells were washed three times, and a Cell Cycle Analysis Kit (Beyotime, Shanghai, China) was used for propidium iodide (PI) staining.

For apoptosis analysis, all cells were treated with 0.5 mM H_2_O_2_ for 4 h to stimulate apoptosis. An Annexin VAPC/7-AAD Apoptosis Detection Kit (KeyGEN, Jiangsu, China) was used for Annexin V-APC and 7-AAD staining according to the manufacturer’s protocol. Finally, the cell cycle distribution percentage and the apoptotic rate were analyzed using BD FACSCanto II (BD Biosciences, San Jose, CA, USA).

### Animal models

Four-week-old male BALB/c nude mice were purchased from the Animal Center of Nanjing Medical University (Nanjing, China) for subcutaneous tumor formation and liver metastasis model. For the xenograft model, 10^6^ DLD-1 cells transfected with sh-circ-GALNT16#1 and RKO cells transfected with circ-GALNT16 as well as corresponding control cells were resuspended in 1 mL phosphate-buffered saline (PBS) and subcutaneously injected into the left and right armpits of the mice, respectively. The volume of each tumor was calculated every 5 days since the tumor became macroscopic. All the mice were sacrificed 30 days after the subcutaneous injection, and the xenograft tumors were weighed and subjected to immunohistochemistry (IHC).

A total of 10^6^ cells suspended in 20 µL PBS were injected into the distal tip of the spleen of mice to establish the metastatic model. The liver tissues were dissected and embedded in paraffin for hematoxylin and eosin (H&E) staining 6 weeks later. All the animal experiments were ratified by the Committee on the Ethics of Animal Experiments of Nanjing Medical University.

### IHC

IHC was performed as described previously [21]. All the antibodies used are listed in Table S4.

### RNA and protein isolation of nuclear and cytoplasmic fractions

Nuclear and cytoplasmic fractions of CRC cells were separated with a PARIS™ kit (AM1556; Thermo Fisher Scientific). RNA was isolated according to the protocol and analyzed by qRT-PCR. U6, 18 S, and GAPDH were used as internal controls. The isolated nuclear protein was prepared for the subsequent pulldown assay.

### Fluorescence *in situ* hybridization and immunofluorescence

Circ-GALNT16-specific Cy3-labeled probe was used to detect the subcellular localization of circ-GALNT16 in DLD-1 and LoVo cells by using a FISH Kit (RiboBio). Briefly, after the cells were fixed with paraformaldehyde for 10 min and permeabilized for 5 min by using PBS with 0.5 % Triton X-100, a FISH probe hybridized with a preheated hybridization buffer was mixed with cells and incubated overnight at 37 °C. The cells were then washed with 4× sodium citrate buffer containing 0.1 % Tween-20 for 5 min and 1× SSC for 5 min. The cell nucleus was stained with 4,6-diamidino-2-phenylindole (DAPI). The images were obtained using a confocal fluorescence microscope.

For the dual RNA-FISH and immunofluorescence assay, an immunostaining blocking solution (Beyotime) was used for cell blocking for 1 h after incubation with the FISH probe as described above. The cells were then incubated with hnRNPK antibodies overnight and labeled with fluorescent secondary antibodies for 1 h in the dark. Finally, DAPI was used for nuclear staining.

### RNA pulldown assay and mass spectrometry

A biotin-labeled pulldown probe targeting circ-GALNT16 and a control probe were designed and synthesized by Ribobio. The RNA pulldown assay was performed using the Pierce Magnetic RNA-Protein Pull-Down Kit according to the protocol (#20,164; Thermo Fisher Scientific). Mass spectrometry analysis was performed using the elution protein extracted from the RNA pulldown assay. The differential proteins identified by mass spectrometry and the list of RNA-binding proteins (RBPs; http://www.ablife.cc) are shown in Table S5.

### Western blotting

Western blotting (WB) was performed as reported previously [19]. The antibodies are shown in Table S4.

### RIP and coimmunoprecipitation assay

The RNA immunoprecipitation (RIP) assay was performed using a RIP Kit (Millipore, Burlington, MA, USA). In brief, 5 µg anti-hnRNPK or anti-FLAG antibodies and magnetic beads were mixed and incubated with cells lysed with RIP lysis buffer supplemented with protease and RNase inhibitors overnight at 4 °C. The immunoprecipitated RNA was obtained for qRT-PCR after digestion with proteinase K buffer.

Coimmunoprecipitation assay was performed with an IP/Co-IP Kit (#88,828; Thermo Fisher Scientific) to determine the interactions of hnRNPK with p53 and SENP2. The detailed procedures were performed as previously described [22].

### SUMOylation modification analysis and UV irradiation

The treated cell lysate was diluted 20-fold with a lysis buffer containing 20 mM Tris-HCl (pH = 8.0), 150 mM NaCl, 2 mM NEM, 1× protease inhibitor cocktail, and 0.2 % Triton X-100. The lysate was incubated with anti-hnRNPK antibodies for 4 h at 4 °C. The lysate was mixed and incubated with protein A/G-Sepharose (sc-2003; Santa Cruz Biotechnology, USA) overnight at 4 °C. The resins were washed with a lysis buffer containing 1 % Triton X-100 for three times and boiled in 50 µL SDS sample buffer for 10 min. Finally, WB was performed with supernatants using anti-hnRNPK and anti-SUMO1 antibodies.

The UV treatment was performed as previously reported. Breifly, cells were treated with UV at 50-70 % confluency by UV phototherapy instruments (Sigma, Shanghai, China), whereas irradiation dose (10 J/m2)was measured with irradiance monitor (Sigma, Shanghai, China). After recovery in medium with 10 % fetal bovine serum for 6 h, cells were harvestd for SUMOylation modification analysis.

### RNA sequencing assay

RNA-seq libraries were prepared after RNA-Seq was performed in RKO cells transfected with a circ-GALNT16 overexpression lentivirus and control cells. First, RNA samples that had passed quality inspection were developed with a starting amount of 1 µg and subjected to RNA integrity check by an Agilent 4200 TapeStation. The key steps were as follows: (1) RNA was purified with polyA Oligo magnetic beads; (2) first-strand cDNA synthesis was performed with random hexamer primers; (3) RNA degradation was performed with RNase H, and second-strand cDNA synthesis was carried out using DNA polymerase I; (4) end repair was performed for the double-stranded cDNA fragments, and a single “A” base was added at the 3ʹ-end of each strand; (5) ligation with special sequencing adapters was performed, and PCR amplification was conducted. Finally, a HiSeq 2000 system on Pair End (Illumina, San Diego, CA, USA) was used to sequence the purified cDNA. The mRNA sequencing results are shown in Table S6.

### Chromatin immunoprecipitation assays

A chromatin immunoprecipitation (ChIP) Kit (CST, #56,383; Danvers, MA, USA) was used for the ChIP assay according to the manufacturer’s protocol. Briefly, chromatin fragments were sheared to 200- to 1,000-bp size by using ChIP sonication lysis buffers. The beads-antibody complexes were then incubated with sheared crosslinked chromatin. After the DNA was purified, qRT-PCR was performed using ChIP primers. The sequences of ChIP primer are listed in Table S3.

### Statistical analysis

Each experiment was repeated at least three times. All statistical analyses were performed with GraphPad Prism software (La Jolla, CA, USA) and SPSS 13.0 software (Chicago, IL, USA). The student’s *t*-test was performed to analyze the difference between two samples, while ANOVA was used for tests among more than two groups. Pearson’s correlation analysis was performed to estimate the correlation between circ-GALNT16 and Serpine1. The chi-square test was used to analyze the correlation between circ-GALNT16 expression in tumor tissues and relative clinicopathological data. Overall survival (OS) rates were estimated using Kaplan-Meier curves. The significance threshold of each test was set at 0.05.

## Results

### Circ-GALNT16 is downregulated in CRC tissues and correlates with good prognosis

**Fig. 1:**
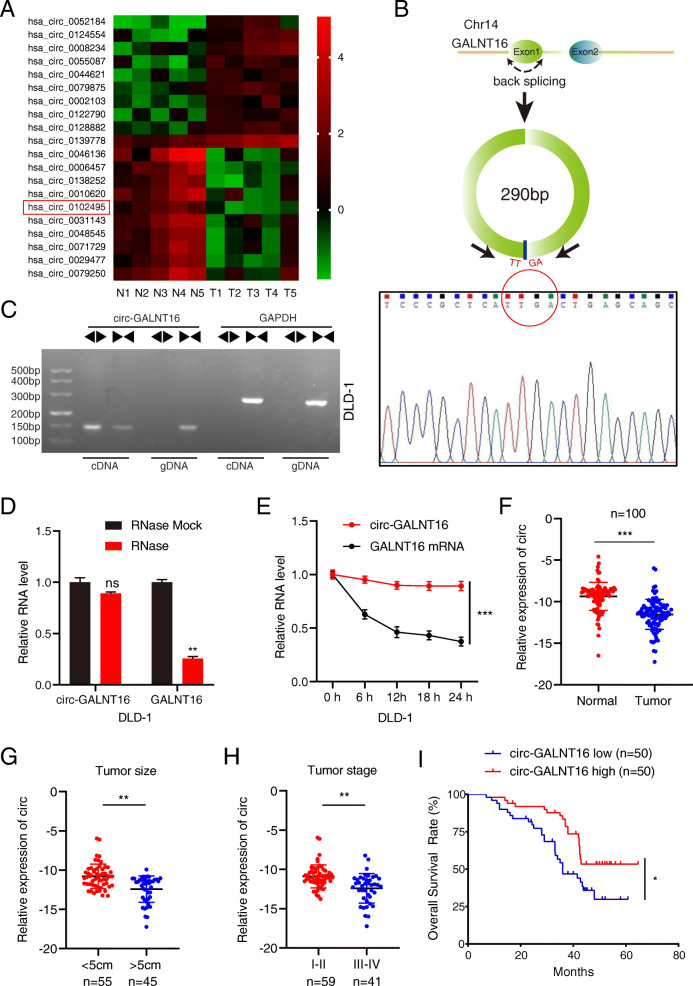
Circ-GALNT16 validation and expression in CRC cells and tissues. **a** Heatmap of top ten upregulated and downregulated expression circRNAs between CRC tissues and adjacent normal tissues according to the circRNA microarray. **b** The schematic illustration showed the back splicing of circ-GALNT16, and sanger sequence validated the splicing site. **c** PCR and agarose gel electrophoresis confirmed the circular formation of circ-GALNT16, using divergent and convergent primers in gDNA and cDNA of DLD-1. GAPDH was used as a negative control. **d, e.** Circ-GALNT16 and linear GALNT16 expression levels were detected after RNase R or actinomycin D treatment in DLD-1. **f.** Relative circ-GALNT16 expression in 100 CRC tissues and matched adjacent normal tissues. **g-h.** Relative expression of circ-GALNT16 in different tumor size and tumor stage groups. **i.** Kaplan-Meier plots of the overall survival of CRC patients with high (*n* = 50) and low (*n* = 50) levels of circ-GALNT16. All data are presented as the means ± SD of three independent experiments. ^ns^*p* > 0.05, *p < 0.05, **p < 0.01, ***p < 0.001

To determine pivotal circRNAs involved in CRC progression, a circRNA microarray was developed using matched CRC and adjacent normal tissues from 5 patients, and the top 20 upregulated and downregulated circRNAs are shown by heatmap (Fig. 1a). We selected the top 10 differentially expressed circRNAs (*P* < 0.01) for further qRT-PCR verification in 20 paired tumor tissues (Fig. S1a). Circ-GALNT16 (circBase ID: hsa_circ_0102495) was highly downregulated in CRC tissues. Therefore, circ-GALNT16 was chosen for our subsequent in-depth study. Circ-GALNT16 is generated from exon1 of the GALNT16 gene located on chromosome 14 and is 290 nucleotides in length according to the circBase annotation. Sanger sequencing was performed to validate the back-splicing of circ-GALNT16 (Fig. 1b). To confirm the circular formation of circ-GALNT16, both convergent and divergent primers were designed to amplify the linear and back-splicing products. The results demonstrated that the convergent primers for both circ-GALNT16 and GAPDH could amplify products of expected size from both cDNA and genomic DNA (gDNA). Only divergent primers for circ-GALNT16, but not for GAPDH, could amplify a PCR product from cDNA, but not from gDNA (Fig. 1c and Fig. S2a). In addition, qRT-PCR confirmed that circ-GALNT16 was more resistant to RNase R and actinomycin D treatment than GALNT16 mRNA in DLD-1 and LoVo cells (Fig. 1d, e and Fig. S2b, c). These results indicated that circ-GALNT16 was a circular, not linear structure. Circ-GALNT16 was remarkably downregulated in 100 CRC tissues compared to that in paired adjacent normal tissues (Fig. 1f and Fig. S2d). Correspondingly, the expression of circ-GALNT16 in normal colorectal epithelial cells NCM460 was significantly higher than that in CRC cell lines (Fig. S2e). The correlation between circ-GALNT16 expression level and clinical subgroups was analyzed. Circ-GALNT16 expression was negatively correlated with tumor size, tumor stage, and lymph node metastasis (Fig. 1g, h and Fig. S2f). To further investigate the link between circ-GALNT16 expression level and clinical characteristics, the specimens were separated into two groups according to the median circ-GALNT16 expression level. As shown in Table 1, significant differences were noted in tumor size, tumor stage, and lymph node metastasis between the high and low circ-GALNT16 expression groups, but there were no differences in sex, age, and carcinoembryonic antigen (CEA) level. In addition, lower circ-GALNT16 expression in tumor tissues was correlated with shorter OS (Fig. 1i). All these clinical data suggested that circ-GALNT16 was downregulated in CRC and may function as a potential diagnostic and prognostic biomarker for CRC.

**Table 1:** Relevance analysis of circ-GALNT16 expression in CRC patients (*n* = 100)

Variables	All patients	circ-GALNT16		*P* value
		Low	High	
All Cases	100	50	50	
Age (years)				
<60	35	16	19	0.53
≥60	65	34	31	
Gender				
Female	41	18	23	0.31
Male	59	32	27	
Tumor size (cm)				
<5	55	19	36	**0.001**
≥5	45	31	14	
Tumor stage				
Stage I+II	59	22	37	**0.002**
Stage III+IV	41	28	13	
Lymph node metastasis				
No	61	23	38	**0.002**
Yes	39	27	12	
Distant metastasis				
No	93	47	46	>0.99
Yes	7	3	4	
CEA (ng/ml)				
<4.70	46	25	21	0.42
≥4.70	54	25	29	

NOTE: *CEA* carcinoembryonic antigen

All data are presented as the means ± SD of three independent experiments. *P* ≤ 0.05 was considered significant. The bold type represents *P* values smaller than 0.05

### Circ-GALNT16 inhibits the proliferation and metastasis of CRC cells *in vitro* and *in vivo*

**Fig. 2:**
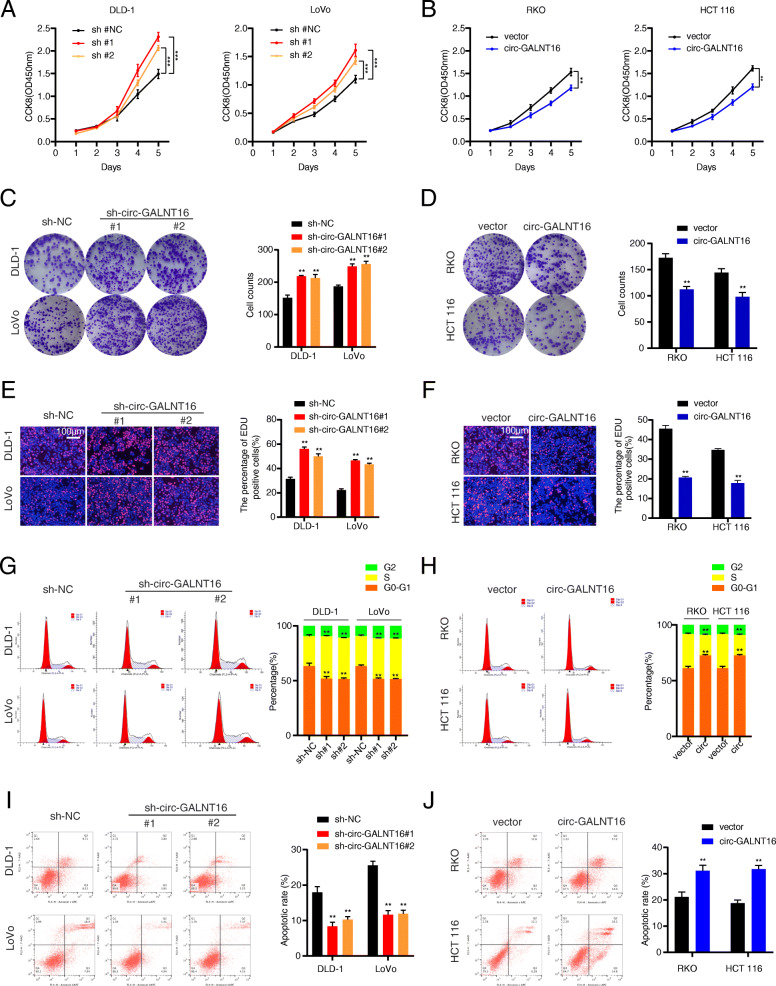
Circ-GALNT16 suppresses the proliferation of CRC cells in vitro. **a, b.** CCK8 assays were applied to determine the growth curves of circ-GALNT16 knockdown or overexpression cells. **c, d.** Colony formation assays were performed to evaluate cell proliferation ability. **e, f.** EdU assays were performed to assess the cell proliferation ability. **g, h.** Cell cycle distributions were detected by flow cytometry in circ-GALNT16 knockdown or overexpression cells. **i, j.** The apoptotic rates were performed and analyzed after cells were treated with 0.5mM H_2_0_2_ for 4 h. All data are presented as the means ± SD of three independent experiments. **p < 0.01, ***p < 0.001

**Fig. 3:**
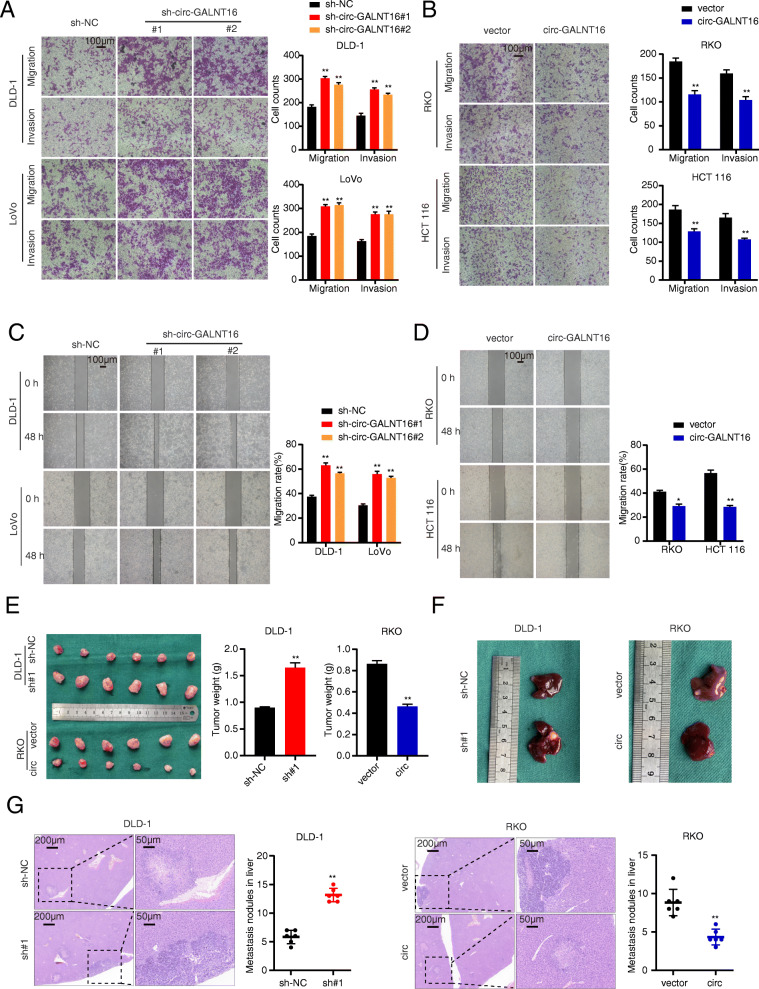
Effects of circ-GALNT16 on the proliferation, migration, and invasion *in vitro* and *vivo*. **a, b**. Transwell assays were applied to evaluate the migration and invasion abilities of CRC cells. **c, d.** Cell migration ability was assessed by wound healing assay. **e.** Representative photographs of subcutaneous xenograft tumors were obtained from nude mice, and the tumor weights were measured. **f, g.** Representative images and HE staining of liver metastatic tumors. All data are presented as the means ± SD of three independent experiments. *p < 0.05, **p < 0.01

Because of the comparatively high expression of circ-GALNT16 in LoVo and DLD-1 cells, three shRNAs against circ-GALNT16 were transfected into LoVo and DLD-1 cells. A circ-GALNT16 overexpression lentivirus was transfected into RKO and HCT 116 cells because of their relatively low expression of circ-GALNT16 in order to elucidate the roles of circ-GALNT16 in CRC proliferation and metastasis. The efficiency of circ-GALNT16 knockdown and overexpression is confirmed by qRT-PCR. The expression of GALNT16 mRNA remained unchangeable in circ-GALNT16 knockdown or overexpressing cells (Fig. S3a, b). Both sh-circ-GALNT16#1 and sh-circ-GALNT16#2 were chosen for the subsequent cell phenotype assays. CCK-8, colony formation, and EdU assays demonstrated that circ-GALNT16 silencing excessively promoted cell proliferation in LoVo and DLD-1 cells, while circ-GALNT16 overexpression caused a completely opposite phenomenon in RKO and HCT 116 cells (Fig. 2a-f). Flow cytometric assay of cell cycle distribution demonstrated that the knockdown of circ-GALNT16 facilitated the G1 to S transition. However, the overexpression of circ-GALNT16 dramatically increased the number of cells in G0/G1 phase, along with a remarkable decrease in S phase cells (Fig. 2g, h). Apoptosis assay showed that the CRC cells transfected with shRNAs had lower apoptotic rates than the control group. Conversely, the apoptotic rates remarkably increased when circ-GALNT16 was overexpressed in RKO and HCT 116 cells (Fig. 2i, j). Additionally, Transwell and scratch wound healing assays confirmed that circ-GALNT16 depletion prominently enhanced the migration and invasion ability of CRC cells, while circ-GALNT16 overexpression impaired this ability (Fig. 3a-d).

DLD-1 cells stably transfected with sh-circ-GALNT16#1 and RKO cells stably transfected with circ-GALNT16, along with their relative control group, were subcutaneously injected into nude mice to investigate the regulatory effect of circ-GALNT16 on CRC cell proliferation *in vivo*. The results showed that tumors generated from circ-GALNT16-overexpressing cells had less tumor volume and weight than the control group, while circ-GALNT16 depletion had an opposite effect (Fig. 3e and Fig. S3c). Furthermore, Ki-67, C-myc, and Serpine1 expression levels were increased in the circ-GALNT16 knockdown group and decreased in the circ-GALNT16 overexpression group as measured by IHC (Fig. S3d). The tumor metastasis assay confirmed that the depletion of circ-GALNT16 resulted in more liver metastatic nodules. In contrast, circ-GALNT16 overexpression caused less liver metastasis than that observed for the control group (Fig. 3f, g). Collectively, all these results showed that circ-GALNT16 inhibited the proliferation and metastasis of CRC cells *in vitro* and *in vivo*.

### Circ-GALNT16 suppresses the progression of CRC by specifically binding to the KH3 domain of hnRNPK

**Fig. 4:**
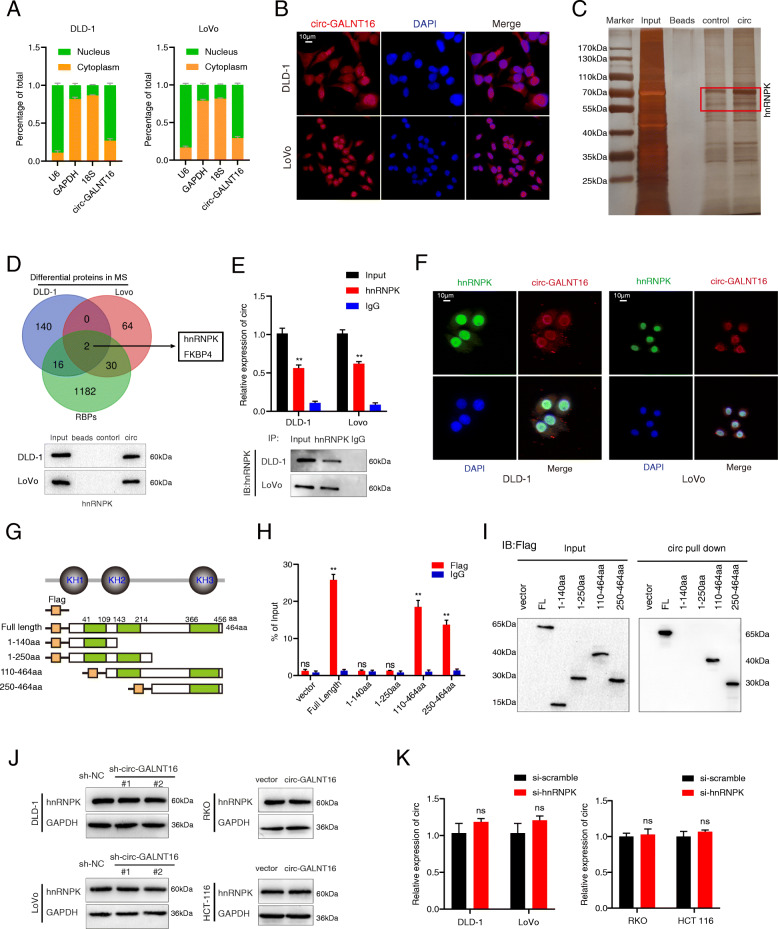
Circ-GALNT16 is mainly localized in the nucleus and binds to the KH3 domain of hnRNPK. **a, b.** Subcellular fractionation and FISH assays indicated that circ-GALNT16 was predominately localized in the nucleus of CRC cells. **c-e.** RNA pulldown assay followed by silver staining, MS, and RIP assay indicated that circ-GALNT16 specifically interacted with hnRNPK. **f.** Dual RNA FISH and immunofluorescence assays showed that circ-GALNT16 and hnRNPK colocalized in the nucleus of DLD-1 and LoVo. **g.** Functional domain and truncated mutation annotation of hnRNPK. **h-i.** RIP assay and RNA pulldown assay confirmed that circ-GALNT16 characteristically interacted with the KH3 domain of hnRNPK. **j.** The protein level of hnRNPK in circ-GALNT16 knockdown and overexpression cells. **k.** The circ-GALNT16 expression level in hnRNPK silencing cells.^ns^*p* > 0.05, **p < 0.01

To determine the tumor suppression mechanisms of circ-GALNT16 in CRC, we first examined the subcellular location of circ-GALNT16 in CRC cells. Subcellular fractionation assay and fluorescent *in situ* hybridization (FISH) assay showed that circ-GALNT16 was predominantly located in the nucleus of CRC cells (Fig. 4a, b and Fig. S3e). Next, we performed the biotin-labeled RNA pulldown assay by using a circ-GALNT16-specific probe and a control probe in DLD-1 and LoVo cells, followed by silver staining to identify the potential protein partner of circ-GALNT16 (Fig. 4c). Mass spectrometry revealed that the circ-GALNT16 probe group contained 156 and 96 differential proteins in DLD-1 and LoVo cells, respectively. After overlapping with RBPs, two candidates were selected from the protein partner of circ-GALNT16: heterogeneous nuclear ribonucleoprotein K (hnRNPK) and FKBP prolyl isomerase 4 (FKBP4). Considering the nuclear location of circ-GALNT16, we chose hnRNPK as the potential partner of circ-GALNT16, and WB verified the results of mass spectrometry (Fig. 4d and Fig. S4a). The hnRNPK was reported to function as a DNA- and RNA-binding protein and to regulate a large number of biological processes and cancer pathogenesis [13]. Additionally, previous studies showed that hnRNPK could influence CRC progression by interacting with noncoding RNA [23, 24]. Next, hnRNPK silencing cells were constructed (Fig. S4b). The RNA pulldown assay was then performed again with the nucleoprotein of DLD-1 cells (Fig. S4c). Additionally, the RIP assay showed that hnRNPK could specifically enrich circ-GALNT16 (Fig. 4e). Dual RNA-FISH and immunofluorescence assay showed the co-localization of circ-GALNT16 and hnRNPK (Fig. 4f). We designed four truncated hnRNPK plasmids aiming at three K homology (KH) domains that mediated nucleic acid binding to determine which domain circ-GALNT16 interacted with. Protein domain mapping and RIP assay showed that circ-GALNT16 interacted with the KH3 domain of hnRNPK (Fig. 4g-i). CCK8 and Transwell assays also indicated that circ-GALNT16 depletion could not affect the proliferation and metastatic ability of CRC cells while hnRNPK was knocked down (Fig. S4d, e). The mRNA and protein levels of hnRNPK did not alter in circ-GALNT16 knockdown or overexpressing cells (Fig. 4j and Fig. S4f). The expression of circ-GALNT16 also remained intact while hnRNPK was knocked-down (Fig. 4k). Collectively, circ-GALNT16 restrained CRC progression by interacting with the KH3 domain of hnRNPK.

### Circ-GALNT16 enhances hnRNPK-p53 interaction by inhibiting SENP2-mediated hnRNPK deSUMOylation, and hnRNPK facilitates the nuclear accumulation of circ-GALNT16

**Fig. 5:**
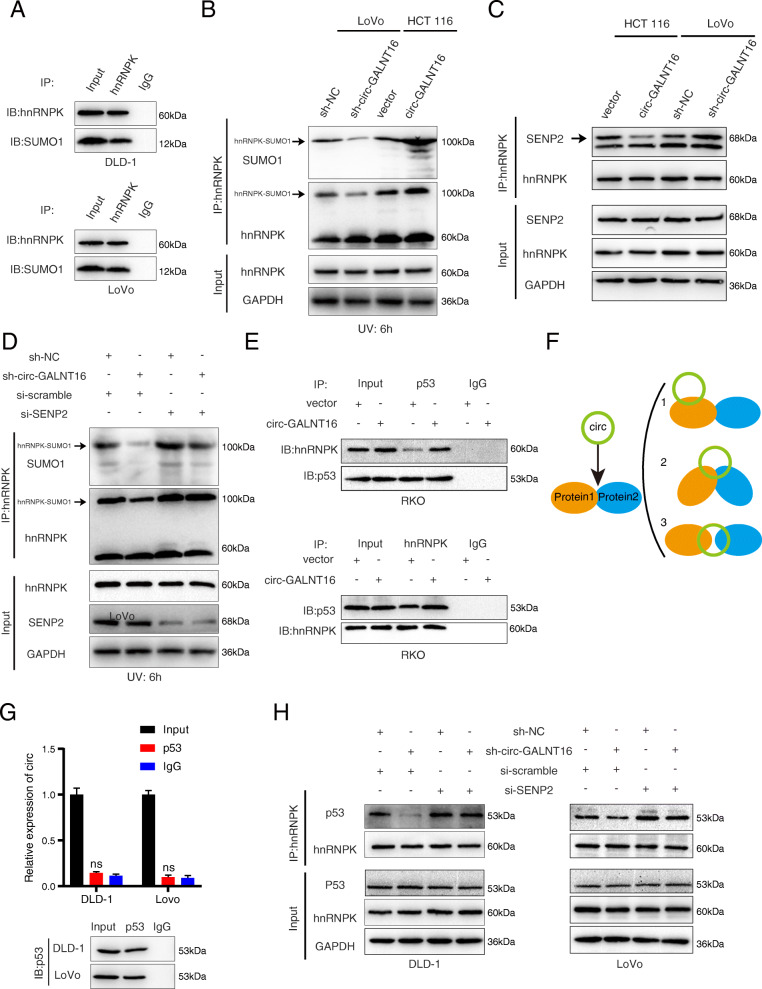
Circ-GALNT16 promotes the interaction between hnRNPK and p53 via inhibiting SENP2-mediated deSUMOylation. **a** Co-IP assay was performed in protein lysate pulled down by circ-GALNT16 specifical probe using anti-hnRNPK. **b** SUMOylation modification analysis was performed to identify the levels of hnRNPK SUMOylation in circ-GALNT16 knockdown and overexpression cells at 6 h after UV stimulation. **c** The co-IP assay was performed between SENP2 and hnRNPK in circ-GALNT16 silencing and overexpression cells. **d** SUMOylation modification analysis to explore the levels of hnRNPK SUMOylation in circ-GALNT16 knockdown and SENP2 knockdown cells at 6 h after UV stimulation. **e** The co-IP assay elucidated that circ-GALNT16 could mediate the interaction between hnRNPK and p53. **f** Three modes of circRNA-protein interactions. **g** RIP assay showed p53 did not interact with circ-GALNT16 directly. **h** Co-IP assay between p53 and hnRNPK applied in circ-GALNT16 and SENP2 depletion cells. All data are presented as the means ± SD of three independent experiments. ^ns^*p* > 0.05, **p < 0.01

Given that circ-GALNT16 specifically interacts with hnRNPK, we next investigated how circ-GALNT16 restrained CRC progression through hnRNPK. Because the protein levels of hnRNPK were not changed in circ-GALNT16 knockdown or overexpressing cells, we assumed that circ-GALNT16 could influence some kind of posttranslational modification of hnRNPK by binding to it. Previous studies have reported that the KH3 domain was the site of hnRNPK SUMOylation and that noncoding RNA could regulate the SUMOylation of hnRNPK in hepatocellular carcinoma. SUMOylation is a vital posttranslational modification of hnRNPK involved in transcriptional regulation, DNA repair, etc. [14, 25]. Therefore, we wondered whether circ-GALNT16 could regulate the SUMOylation modification of hnRNPK. A co-IP assay was performed with the protein lysate pulled down by the circ-GALNT16-specific probe, and the results indicated that circ-GALNT16 might mediate the interaction between hnRNPK and SUMO1 (Fig. 5a). Next, SUMOylation modification analysis was performed. The results showed that knockdown of circ-GALNT16 could decrease the SUMOylation modification of hnRNPK at 100 kDa, which was the theoretical molecular weight of hnRNPK conjugated with SUMO1 (Fig. S5a). SUMOylation is easily lost after lysis due to the high activity of SUMO isopeptidase, which may lead to the expression level of SUMOylation of hnRNPK seems little [26]. It has been reported that ultraviolet exposure could accumulate the SUMOylation of hnRNPK and impair the interaction between hnRNPK and SUMO-specific peptidase 2 (SENP2) at 6 h after UV stimulation [16]. Cells were harvested at 6 h after UV exposure for SUMOylation modification analysis, and the results showed that circ-GALNT16 could enhance the SUMOylation modification of hnRNPK with UV stimulation (Fig. 5b). SENP2 can antagonistically regulate hnRNPK SUMOylation in HeLa and HCC cells [16, 25]. However, it remains unclear whether SENP2 could facilitate the deSUMOylation of hnRNPK in CRC cells. A co-IP assay confirmed that SENP2 could bind to hnRNPK in CRC cells (Fig. S5b). We then found that circ-GALNT16 overexpression could impair the interaction between SENP2 and hnRNPK, and circ-GALNT16 knockdown enhanced this interaction (Fig. 5c). Similar results were presented in cells with UV exposure (Fig. S5c). SENP2 knockdown cells were constructed (Fig. S5d). SUMOylation modification analysis revealed that circ-GALNT16 enhanced the SUMOylation of hnRNPK through SENP2 (Fig. 5d and Fig. S5e). These data indicated that circ-GALNT16 could weaken the deSUMOylation modification of hnRNPK through binding to hnRNPK and blocking the interaction between hnRNPK and SENP2.

Several studies have reported that the SUMOylation of hnRNPK could enhance the interaction between hnRNPK and p53, which plays a major role in cancer progression [16, 17, 25]. A co-IP assay indicated that circ-GALNT16 could enhance the interaction between hnRNPK and p53 (Fig. 5e). A previous review summarized three approaches through which circRNAs regulate interactions of two proteins: (1) regulating the function of one protein but not binding to the other protein; (2) forming a scaffold and cementing their interaction by binding to both of them; and (3) acting as a dissociator between two proteins (Fig. 5f) [27]. Because circ-GALNT16 did not physically bind to p53, we hypothesized that circ-GALNT16 regulated the interaction between hnRNPK and p53 by enhancing the SUMOylation of hnRNPK, rather than acting as a scaffold between hnRNPK and p53 (Fig. 5g and Fig. S5f). SENP2 silencing could abolish the dissociation between hnRNPK and p53 and inhibit the proliferation of CRC cells caused by knockdown of circ-GALNT16 (Fig. 5h, and Fig. S5g). Collectively, circ-GALNT16 enhanced the interaction between hnRNPK and p53 by blocking SENP2-mediated deSUMOylation of hnRNPK.

After we proved that circ-GALNT16 could specifically interact with hnRNPK and hnRNPK was reported to play a pivotal role in RNA transcription and alternative splicing [28–30], we wondered whether hnRNPK could regulate the transcription or splicing of circ-GALNT16. Neither circ-GALNT16 nor GALNT16 showed a significant variation in their expression while hnRNPK was knocked down, indicating that hnRNPK did not participate in the transcription or splicing of circ-GALNT16 (Fig. S6a). A previous study reported that hnRNPK played an important role in driving long noncoding RNA nuclear enrichment [31]. Considering that most circular RNAs are mainly localized in the cytoplasm and function as miRNA sponge, but circ-GALNT16 had nuclear localization, we again performed RNA FISH and subcellular fractionation assay in hnRNPK-depleted cells; the results showed that circ-GALNT16 tended to accumulate in the cytoplasm of cells with low hnRNPK expression (Fig. S6b, c). These results indicated hnRNPK might contribute to the nuclear localization of circ-GALNT16.

### Circ-GALNT16 impairs the expression of Serpine1 in CRC

**Fig. 6:**
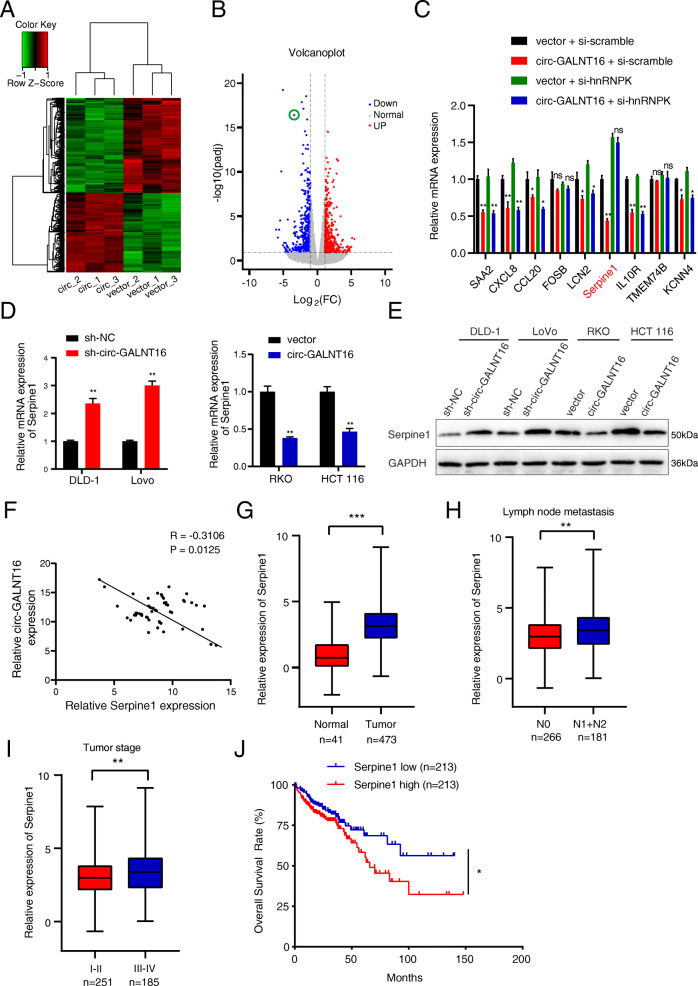
Serpine1 is downregulated by circ-GALNT16 and has a connection with CRC progression. **a, b.** Heatmap and scatter diagram showed the RNA-seq result in circ-GALNT16 overexpression and control RKO cells. **c.** Several candidates were validated by qRT-PCR. **d, e.** The mRNA and protein levels of Serpine1 in circ-GALNT16 knockdown and overexpression cells. **f.** Correlation analysis between circ-GALNT16 and Serpine1. (*R* = -0.3106, *P* = 0.0125) **g-j.** Serpine1 expression in CRC and the correlation with relative clinical features analysis from TCGA. All data are presented as the means ± SD of three independent experiments. ^ns^*p* > 0.05, *p < 0.05, **p < 0.01, ***p < 0.001

Given that circ-GALNT16 could enhance the level of the hnRNPK-p53 transcriptional complex, RNA-seq was performed in circ-GALNT16-overexpressing and related control RKO cells to determine how circ-GALNT16 regulated CRC progression at the transcriptional level (Fig. 6a). A total of 265 genes were downregulated, and 221 genes were upregulated (Fig. 6b). The expression analysis of the top 30 differential genes in CRC tissues was performed using the public GEPIA dataset based on the TCGA (http://gepia.cancer-pku.cn/index.html). Nine genes were selected as target candidates of circ-GALNT16. The results of qRT-PCR showed that only Serpine1 could be downregulated by circ-GALNT16 overexpression but exhibited no significant difference when hnRNPK was silenced (Fig. 6c). Coincidentally, published reports and the KEGG (Kyoto Encyclopedia of Genes and Genomes) pathway database indicated that Serpine1 could be regulated by p53 [32]. The mRNA and protein levels of Serpine1 in circ-GALNT16 knockdown and overexpressing cells revealed that Serpine1 could be antagonistically regulated by circ-GALNT16 (Fig. 6d, e). The expression of Serpine1 in 52 tumor tissues was detected, and the results showed that Serpine1 mRNA level was negatively correlated by circ-GALNT16 expression (Fig. 6f). We then analyzed the correlation between Serpine1 expression and relative clinical features from TCGA. Serpine1 was upregulated in CRC tissues and positively correlated with advanced tumor stage, lymphoid metastasis, and lower OS rate (Fig. 6g-j). These features were contrary to the characteristics of circ-GALNT16.

### Circ-GALNT16 suppresses the proliferation and metastasis of CRC by downregulating Serpine1

**Fig. 7:**
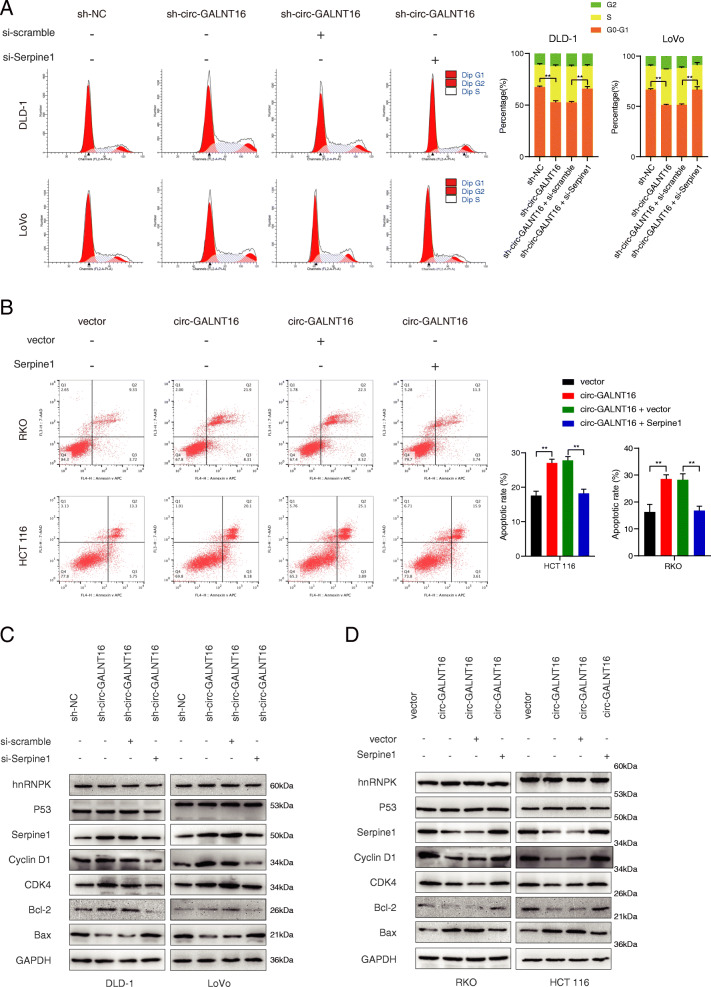
Circ-GALNT16 regulates cell cycle and apoptosis through Serpine1. **a** Cell cycle assays were performed in sh-circ-GALNT16 and si-Serpine1 co-transfected cells. **b** Cell apoptosis assays were performed in circ-GALNT16 overexpression and Serpine1 overexpression groups. **c-d.** The expression of cell cycle and apoptosis makers (Cyclin D1, CDK4, Bcl-2, and Bax) along with hnRNPK, p53, and Serpine1 were detected by western blot in relatively treated cells. **p < 0.01

Given that circ-GALNT16 could suppress the proliferation and metastasis of CRC and the expression of Serpine1, we hypothesized that circ-GALNT16 might suppress CRC progression by inhibiting Serpine1 expression. First, Serpine1-specific siRNA was transfected into DLD-1 and LoVo cells, and an overexpression plasmid was transfected into RKO and HCT 116 cells (Fig. S7a). Cell proliferation assays showed that Serpine1 silencing could remarkably decrease the proliferation ability of circ-GALNT16 knockdown cells. In contrast, Serpine1 overexpression could promote the proliferation ability of circ-GALNT16-overexpressing cells (Fig. S7b-g). Besides, Cell cycle assays showed that the knockdown of circ-GALNT16 decreased the number of cells in G0/G1 phase along with a remarkable increase in S phase cells, and this effect could be rescued by the knockdown of Serpine1 (Fig. 7a). The overexpression of circ-GALNT16 could impair the G1 to S translation, and Serpine1 overexpression could enhance the translation (Fig. S8a). Cell apoptosis assay demonstrated that the proapoptotic effect of circ-GALNT16 could be weakened by Serpine1(Fig. 7b and Fig. S8b). It has been reported that cyclin D/CDK4 induced G1/S transition, while Bcl-2/Bax regulated cell apoptosis antagonistically [33, 34]. The expression of CDK4, Cyclin D1, Bcl-2, and Bax were determined, and results were consistent with previous cell cycle and apoptosis assays (Fig. 7c, d). Wound healing assay and transwell assays showed that circ-GALNT16 could suppress the metastasis ability of CRC through Serpine1 (Fig. S9a-d). Taken together, these results showed that circ-GALNT16 suppresses the proliferation and metastasis of CRC by downregulating Serpine1.

### Circ-GALNT16 attenuates Serpine1 and enhances the p21 mRNA expression level by regulating the sequence-specific DNA-binding ability of the hnRNPK-p53 complex

**Fig. 8:**
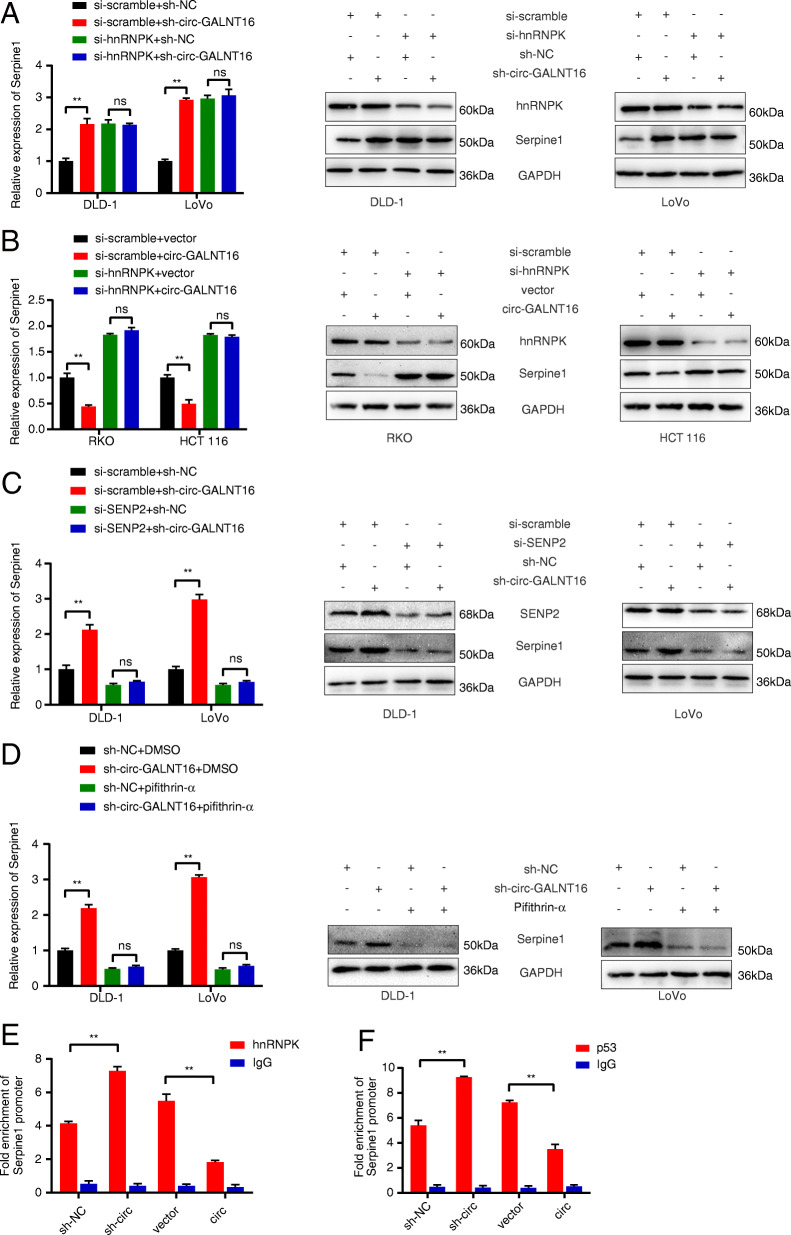
Circ-GALNT16 regulates Serpine1 through the SUMOylation of hnRNPK and p53. **a, b.** The mRNA and protein level of Serpine1 was detected in circ-GALNT16 knockdown and overexpression cells while hnRNPK was silenced. **c.** The expression of Serpine1 was detected in circ-GALNT16 knockdown cells while SENP2 was silenced. **d.** The expression of Serpine1 in circ-GALNT16 silencing cells was detected with the Pifithrin-α (10µM) treatment. **e, f.** HnRNPK and p53 chromatin immunoprecipitation were performed to measure the hnRNPK and p53 enrichment at the promoter region(s) of Serpine1 in DLD-1 and RKO. All data are presented as the means ± SD of three independent experiments. ^ns^*p* > 0.05, **p < 0.01

Given that circ-GALNT16 could enhance the hnRNPK-p53 complex and that Serpine1 was a p53 target gene, we hypothesized that circ-GALNT16 might regulate Serpine1 by enhancing the level of the hnRNPK-p53 complex. First, we observed that the inhibition of Serpine1 caused by circ-GALNT16 could be abolished by hnRNPK silencing (Fig. 8a, b). Moreover, considering that a previous study reported that SUMOylation of hnRNPK could regulate the sequence-specific DNA-binding ability of p53 and exert different effects on the individual p53 target gene, the expression of p21, a representative p53 target antioncogene, was detected together with Serpine1. P21 was downregulated by circ-GALNT16 knockdown and upregulated by circ-GALNT16 overexpression, but it showed no remarkable divergence during simultaneous hnRNPK knockdown (Fig. S10a, b). Additionally, the expression of Serpine1 showed no significant difference in circ-GALNT16-depleted cells when SENP2 was silenced, indicating that the SUMOylation of hnRNPK played a vital role in Serpine1 regulation caused by circ-GALNT16 (Fig. 8c). The inhibitory effect of circ-GALNT16 knockdown on p21 mRNA level was abolished by SENP2 silencing (Fig. S10c). Pifithrin-α, a specific inhibitor of p53 transcriptional activity, could rescue the promotion of Serpine1 expression caused by circ-GALNT16 knockdown (Fig. 8d). Pifithrin-α could also abolish the promotion effect of circ-GALNT16 overexpression on the p21 mRNA level (Fig. S10d). ChIP assays revealed that circ-GALNT16 depletion enhanced the binding of hnRNPK and p53 to the promoter region of Serpine1 and decreased the binding to the promoter region of p21; these findings were consistent with previous results. Circ-GALNT16 overexpression had a contrary effect (Fig. 8e, f and Fig. S10e, f). All these results indicated that circ-GALNT16 regulated Serpine1 and p21 expression by modulating the sequence-specific DNA-binding ability of the SENP2-mediated hnRNPK-p53 complex.

## Discussion

**Fig. 9:**
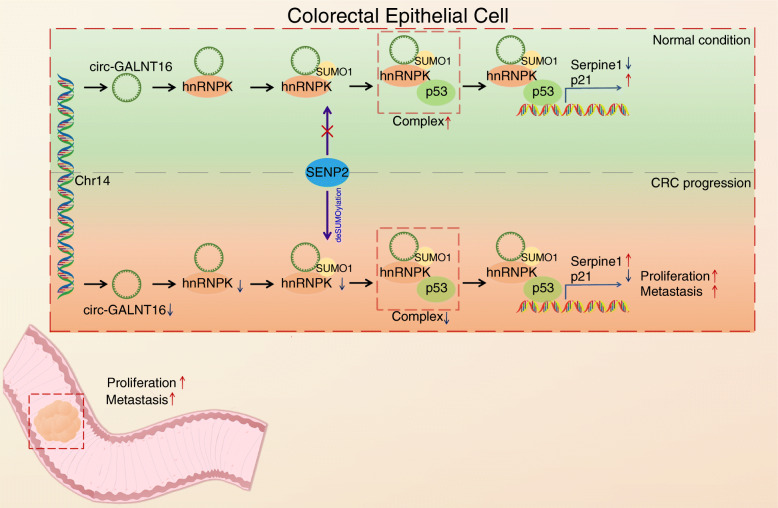
A schematic model for the mechanisms of circ-GALNT16 in CRC

Several studies have indicated that circRNAs, a group of novel noncoding RNAs, can regulate cancer progression through multiple approaches such as functioning as miRNA sponges and interacting with proteins, and thus, they have the potential to function as critical clinical diagnostic and prognostic biomarkers for malignant tumors [35–38]. In the present study, we investigated the role of a novel circRNA, circ-GALNT16, as a tumor suppressor in CRC. Circ-GALNT16 was found to be downregulated in CRC tissues and cells. The low expression of circ-GALNT16 predicted advanced tumor size, tumor stage, lymphatic metastasis, and poor prognosis. Our phenotype experiment and experiments in mice models revealed that circ-GALNT16 suppressed the proliferation, migration, and invasion ability of CRC cell lines. In particular, circ-GALNT16 enhanced hnRNPK-p53 interaction by inhibiting SENP2-mediated deSUMOylation of hnRNPK, which regulated the sequence-specific DNA-binding ability of the hnRNPK-p53 complex (Fig. 9).

Recent studies have shown that circRNAs function as competing endogenous RNAs (ceRNAs) of miRNAs in the cytoplasm [39, 40]. We accidentally found that circ-GALNT16 was predominantly located in the nucleus of CRC cells, thus excluding the possibility of it being a miRNA sponge. The mechanisms of circRNAs with nuclear localization in tumor progression remain obscure and poorly investigated, especially in CRC. In the present study, we found that circ-GALNT16 could specifically bind to the KH3 domain of hnRNPK, which is a multifunctional protein involved in transcriptional regulation, RNA splicing, RNA translocation, and chromatin remodeling [13, 28, 31]. We also found that circ-GALNT16 could enhance the interaction between hnRNPK and p53 by inhibiting SENP2-mediated deSUMOylation of hnRNPK. RNA-seq and qRT-PCR validation indicated that circ-GALNT16 could downregulate the oncogene Serpine1 and upregulate the antioncogene p21 by regulating the sequence-specific DNA-binding ability of the hnRNPK-p53 transcriptional complex. Collectively, our study showed that the circ-GALNT16/hnRNPK/p53 axis could suppress Serpine1 expression in CRC.

Many studies have demonstrated that various posttranslational modifications of hnRNPK, including phosphorylation, ubiquitination, methylation, and SUMOylation, play critical roles in hnRNPK function coactivation [14]. Although several circRNAs have been reported to interact with hnRNPK, the circRNA that regulates the posttranslational modification of hnRNPK has not yet been detected [23, 24, 41]. Moreover, the role of circRNA in the SUMOylation of proteins has not yet been elucidated. In our present study, we first identified circ-GALNT16 that could specifically interact with the KH3 domain of hnRNPK and enhance its SUMOylation modification by inhibiting SENP2 binding to hnRNPK. These results indicated that circRNA could regulate the SUMOylation modification of proteins and provided a new insight for circRNA-based targeting therapy.

RNA-seq and the subsequent qRT-PCR verified that Serpine1 could be downregulated by circ-GALNT16 through the hnRNPK-p53 complex. Several studies have reported that Serpine1 is elevated in multiple tumor tissues, including colorectal cancer, and correlates with poor outcomes. Both p53 and NF-κB have been reported to participate in Serpine1 transcriptional regulation [42–44]. However, the particular mechanisms of Serpine1 remain unclear, especially in CRC. A previous study showed that SUMOylation of hnRNPK induced different effects on the p53 target gene by regulating the sequence-specific DNA-binding ability of p53 [25]. Therefore, after we found that the inhibition of hnRNPK deSUMOylation caused by circ-GALNT16 overexpression could inhibit Serpine1 expression, we proved that circ-GALNT16 overexpression could upregulate the p21 mRNA level through the hnRNPK-p53 complex; this finding was consistent with a previous study [25]. Intriguingly, ChIP assay showed that circ-GALNT16 could attenuate the enrichment of hnRNPK and p53 at Serpine1 promoters but facilitated the enrichment at p21 promoters. The underlying mechanisms for this phenomenon deserve further investigation.

To investigate how circ-GALNT16 mediated the SUMOylation of hnRNPK, we identified SENP2, which was reported to antagonistically mediate the SUMOylation of hnRNPK in HeLa and HCC cells and could interact with hnRNPK in CRC cells [16, 25]. The overexpression of circ-GALNT16 could attenuate the interaction between hnRNPK and SENP2, while circ-GALNT16 knockdown enhanced their interaction. However, we did not determine how circ-GALNT16 mediated the interaction between SENP2 and hnRNPK and the SUMOylation of hnRNPK by specifically binding to the KH3 domain of hnRNPK. A previous study showed that PSTAR could also inhibit the SENP2-mediated deSUMOylation of hnRNPK by interacting with the KH3 domain, which is consistent with our result [25]. Hence, we believe that some correlation might exist between them.

In the present study, we revealed the tumor suppression effect and feasible molecular mechanisms of the novel circRNA circ-GALNT16 in CRC. Circ-GALNT16 was highly downregulated in CRC tissues, which indicated that it might serve as a diagnostic and prognostic biomarker. Our mice models showed that circ-GALNT16 overexpression could inhibit the proliferation and metastatic ability of CRC *in vivo*, thus indicating that circ-GALNT16 might have the potential to act as a therapeutic target for CRC.

## Conclusions

In summary, we identified a novel circRNA, named circ-GALNT16, that was downregulated in CRC and was negatively associated with a poor prognosis. Circ-GALNT16 could suppress the proliferation and aggressiveness of CRC cells and attenuate Serpine1 expression by inhibiting SENP2-mediated hnRNPK deSUMOylation and modulating the sequence-specific DNA-binding ability of the hnRNPK-p53 complex.

## Supplementary Information

**Additional file 1: Figure S1.** The screening of circRNAs. a. The expression of top5 upregulated and downregulated circRNAs in 20 pairs of CRC tissues and relative adjacent normal tissues. All data are presented as the means ± SD of three independent experiments. ^ns^*p* >0.05, *p < 0.05, **p < 0.01, ***p < 0.001.

**Additional file 2: Figure S2.** Circ-GALNT16 validation and expression in CRC tissues and cells. a. PCR and agarose gel electrophoresis confirmed the circular formation of circ-GALNT16, using divergent and convergent primers in gDNA and cDNA of LoVo. b, c. The expression of circ-GALNT16 and linear GALNT16 was detected after RNase R or actinomycin D treatment in LoVo. d. Relative expression of circ-GALNT16 in 100 pairs of CRC and adjacent normal tissues. e. Relative expression of circ-GALNT16 in CRC cell lines and normal epithelial colon cell NCM460. f. Relative expression of circ-GALNT16 in tissue groups with or without lymph node metastasis. All data are presented as the means ± SD of three independent experiments. ^ns^*p* > 0.05 **p < 0.01, ***p < 0.001.

**Additional file 3: Figure S3.** Circ-GALNT16 stably knockdown and overexpression efficiency, and phenotype assays* in vitro* and *vivo*. a. The efficiency of circ-GALNT16 knockdown in DLD-1 and LoVo. The expression of GALNT16 mRNA remained unchangeable. b. The efficiency of circ-GALNT16 overexpression in RKO and HCT 116. The expression of GALNT16 mRNA level was detected at the same time. c. The tumor volumes were measured every 5 days since the subcutaneous tumors were macroscopic. d. Protein levels of Ki67, C-myc, and Serpine1 in the tumor samples were measured by IHC. e. Subcellular fractionation indicated that circ-GALNT16 was predominately localized in the nucleus of CRC cells. All data are presented as the means ± SD of three independent experiments. ^ns^*p *> 0.05 **p < 0.01, ***p < 0.001.

**Additional file 4: Figure S4.** Circ-GALNT16 could specifically interact with hnRNPK in the nucleus and suppress the proliferation and metastasis through binding to hnRNPK. a. MS analysis results of hnRNPK. b. The knockdown efficiency of hnRNPK. c. Pulldown and silver staining performed in nucleoprotein of DLD-1 cells. d, e. CCK8 and transwell showed that circ-GALNT16 suppressed the proliferation and metastasis of CRC by interacting with hnRNPK. f. The mRNA expression level of hnRNPK in circ-GALNT16 knockdown and overexpression cells. All data are presented as the means ± SD of three independent experiments. ^ns^*p* >0.05, *p < 0.05, **p < 0.01.

**Additional file 5: Figure S5. **Circ-GALNT16 promotes the interaction between hnRNPK and p53 via inhibiting SENP2-mediated deSUMOylation. a. SUMOylation modification analysis was performed to identify the levels of hnRNPK SUMOylation in circ-GALNT16 knockdown and overexpression cells. b. The co-IP assay showed SENP2 could interact with hnRNPK in CRC cells. c. The co-IP assay was performed between SENP2 and hnRNPK in circ-GALNT16 silencing and overexpression cells at 6 h after UV stimulation. d. The knockdown efficiency of SENP2. e. SUMOylation modification analysis to explore the levels of hnRNPK SUMOylation in circ-GALNT16 knockdown and SENP2 knockdown cells. f. Pulldown assay showed p53 did not physically interact with circ-GALNT16. g. CCK8 indicated that circ-GALNT16 suppressed the proliferation of CRC by attenuating the deSUMOylation of hnRNPK. All data are presented as the means ± SD of three independent experiments.^ns^*p*>0.05, **p < 0.01.

**Additional file 6: Figure S6.** HnRNPK enhances the nuclear accumulation of circ-GALNT16. a. The mRNA expression level of GALNT16 in hnRNPK depletion cells. b-c. RNA FISH and subcellular fractionation assay again in hnRNPK-depletion cells. All data are shown as mean ± SD of three independent experiments. ^ns^*p* >0.05, **p < 0.01.

**Additional file 7: Figure S7.** Circ-GALNT16 suppresses the proliferation ability of CRC cells through downregulating Serpine1. a. The knockdown and overexpression efficiency of Serpine1. b-g. CCK-8, colony formation, EdU assays were carried out in relatively treated cells targeted circ-GALNT16 and Serpine1. All data are shown as mean ± SD of three independent experiments. *p< 0.05, **p < 0.01.

**Additional file 8: Figure S8.** Circ-GALNT16 regulates cell cycle and apoptosis of CRC cells through downregulating Serpine1. a. Cell cycle assays were performed in circ-GALNT16 overexpression and Serpine1 overexpression groups. b. Cell apoptosis assays were performed in sh-circ-GALNT16 and si-Serpine1 co-transfected cells. **p < 0.01.

**Additional file 9: Figure S9. **Circ-GALNT16 suppresses the metastasis ability of CRC cells through Serpine1. a-d. Transwell and wound healing assays were performed in relatively treated cells targeted circ-GALNT16 and Serpine1. All data are shown as mean ± SD of three independent experiments. *p < 0.05, **p < 0.01.

**Additional file 10: Figure S10. **Circ-GALNT16 promotes p21 mRNA expression level through the SUMOylation of hnRNPK and p53. a, b. The mRNA level of p21 in circ-GALNT16 knockdown and overexpression cells while hnRNPK was silenced. c. The expression of p21 in circ-GALNT16 knockdown cells while SENP2 was silenced. d. The expression of p21 in circ-GALNT16 overexpression cells with the Pifithrin-α (10μM) treatment. e, f. HnRNPK and p53 chromatin immunoprecipitation were performed to measure the hnRNPK and p53 enrichment at the promoter region(s) of p21 in DLD-1 and RKO. All data are shown as mean ± SD of three independent experiments. ^ns^*p *> 0.05, **p < 0.01.

**Additional file 11: Table S1. **The differential expression circRNAs in CRC tissues according to the circRNA microarray.

**Additional file 12: Table S2.** The expression of circ-GALNT16 in 100 pairs of CRC tissues and relative expression in different pathological groups.

**Additional file 13: Table S3.** Primers, shRNAs, siRNAs, and probes used in this study.

**Additional file 14: Table S4.** Primary antibodies information used in this study.

**Additional file 15: Table S5.** Differential proteins identified by mass spectrometry in DLD-1 and LoVo and the list of RNA-binding proteins.

**Additional file 16: Table S6.** The mRNA sequencing results constructed in circ-GALNT16 overexpression and control RKO cells.

## Data Availability

The data supporting the findings of this article is included within this article and its additional files.

## Abbreviations

CRC: Colorectal cancer
circRNA: Circular RNA
GALNT16: Polypeptide N-acetylgalactosaminyltransferase 16
hnRNPK: Heterogeneous nuclear ribonucleoprotein K
SUMO1: Small ubiquitin-related modifier 1
OS: Overall survival rate
GAPDH: Glyceraldehyde 3-phosphate dehydrogenase
SENP2: SUMO specific peptidase 2
Serpine1: Serpin family E member 1
EDU: 5-Ethynyl-2′-deoxyuridine
CCK8: Cell Counting Kit-8
IHC: Immunohistochemistry
qRT-PCR: Quantitative reverse transcription polymerase chain reaction
FISH: Fluorescence in situ hybridization
MS: Mass spectrometry
RBPs: RNA binding proteins
NEM: N-ethylmaleimide
shRNA: :Short hairpin RNA
HCC: Hepatocellular carcinoma
FKBP4: FKBP prolyl isomerase 4
UV: Ultraviolet
