# Supplementary figures and images for: Circ-GALNT16 restrains colorectal cancer progression by enhancing the SUMOylation of hnRNPK
Source: J Exp Clin Cancer Res. 2021 Aug 27;40:272. doi: 10.1186/s13046-021-02074-7 (PMC8400830; doi:10.1186/s13046-021-02074-7)

**A**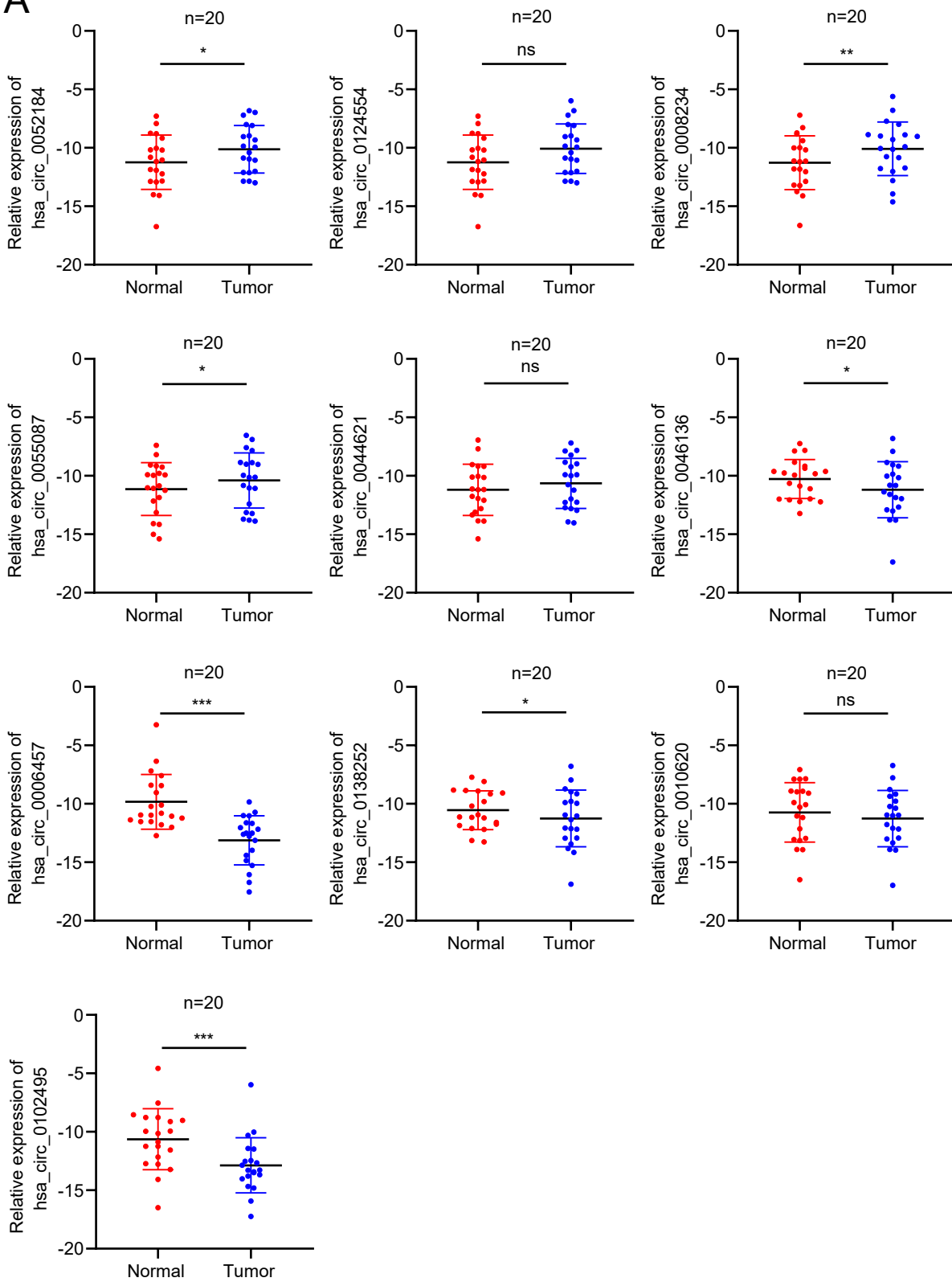

Supplement: Supplementary file 1 — Additional file 1: Figure S1. The screening of circRNAs. a. The expression of top5 upregulated and downregulated circRNAs in 20 pairs of CRC tissues and relative adjacent normal tissues. All data are presented as the means ± SD of three independent experiments. nsp >0.05, *p < 0.05, **p < 0.01, ***p < 0.001. [file 13046_2021_2074_MOESM1_ESM.pdf]

**A**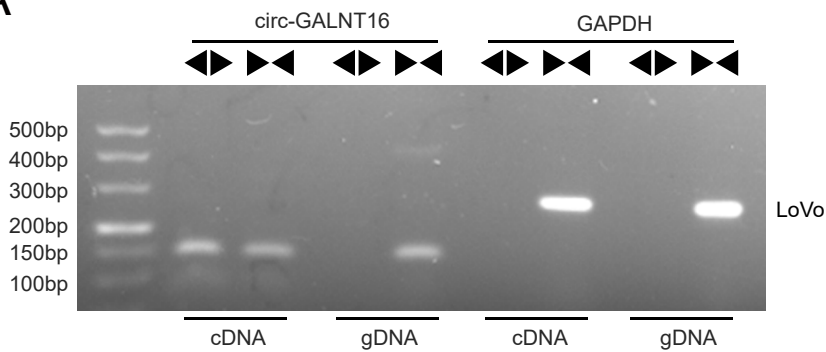**B**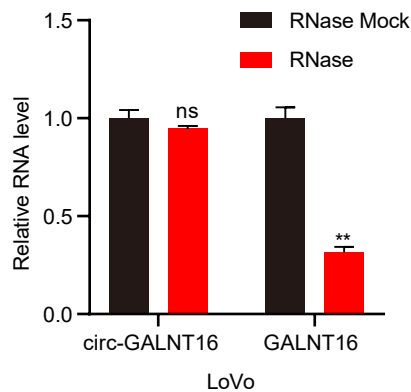**C**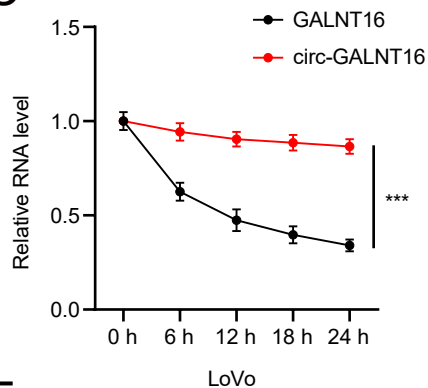**D**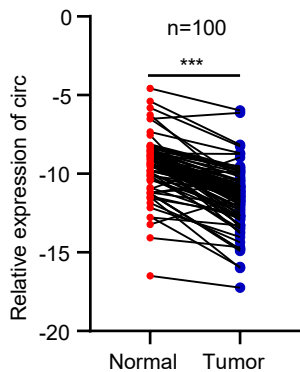**E**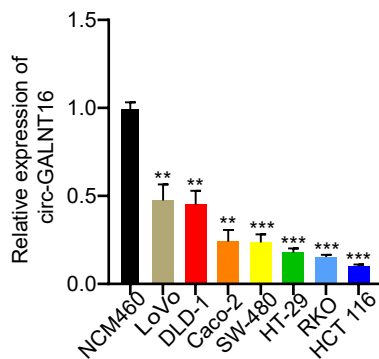**F**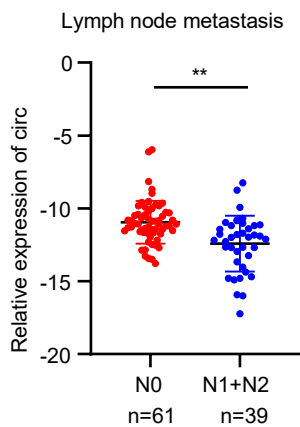

Supplement: Supplementary file 2 — Additional file 2: Figure S2. Circ-GALNT16 validation and expression in CRC tissues and cells. a. PCR and agarose gel electrophoresis confirmed the circular formation of circ-GALNT16, using divergent and convergent primers in gDNA and cDNA of LoVo. b, c. The expression of circ-GALNT16 and linear GALNT16 was detected after RNase R or actinomycin D treatment in LoVo. d. Relative expression of circ-GALNT16 in 100 pairs of CRC and adjacent normal tissues. e. Relative expression of circ-GALNT16 in CRC cell lines and normal epithelial colon cell NCM460. f. Relative expression of circ-GALNT16 in tissue groups with or without lymph node metastasis. All data are presented as the means ± SD of three independent experiments. nsp > 0.05 **p < 0.01, ***p < 0.001. [file 13046_2021_2074_MOESM2_ESM.pdf]

**A**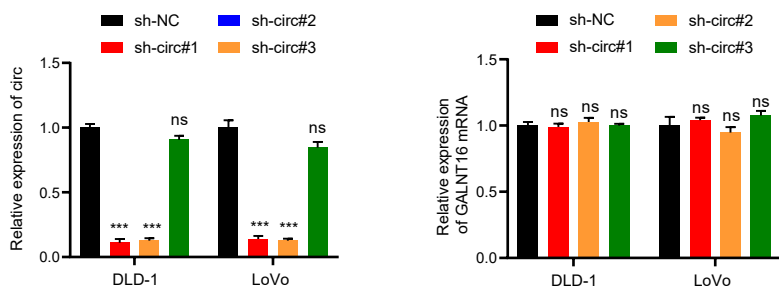**B**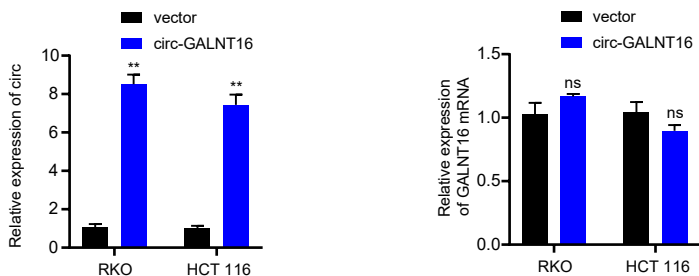**C**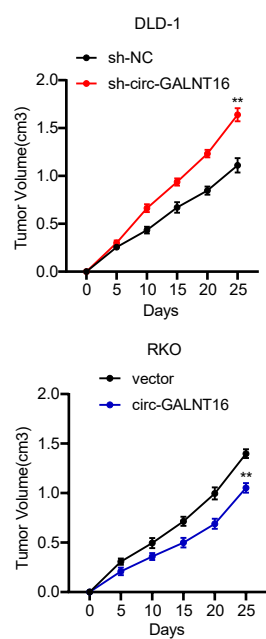**D**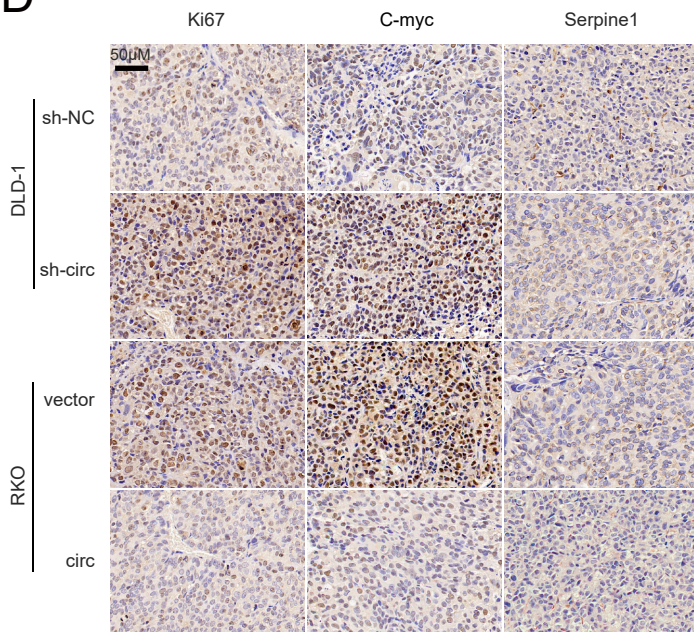**E**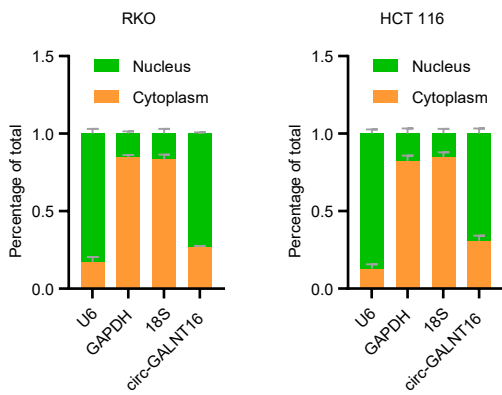

Supplement: Supplementary file 3 — Additional file 3: Figure S3. Circ-GALNT16 stably knockdown and overexpression efficiency, and phenotype assays in vitro and vivo. a. The efficiency of circ-GALNT16 knockdown in DLD-1 and LoVo. The expression of GALNT16 mRNA remained unchangeable. b. The efficiency of circ-GALNT16 overexpression in RKO and HCT 116. The expression of GALNT16 mRNA level was detected at the same time. c. The tumor volumes were measured every 5 days since the subcutaneous tumors were macroscopic. d. Protein levels of Ki67, C-myc, and Serpine1 in the tumor samples were measured by IHC. e. Subcellular fractionation indicated that circ-GALNT16 was predominately localized in the nucleus of CRC cells. All data are presented as the means ± SD of three independent experiments. nsp > 0.05 **p < 0.01, ***p < 0.001. [file 13046_2021_2074_MOESM3_ESM.pdf]

**A**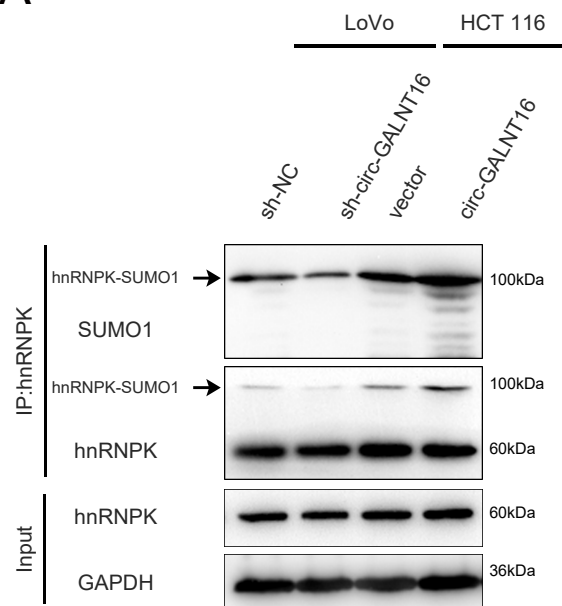**B**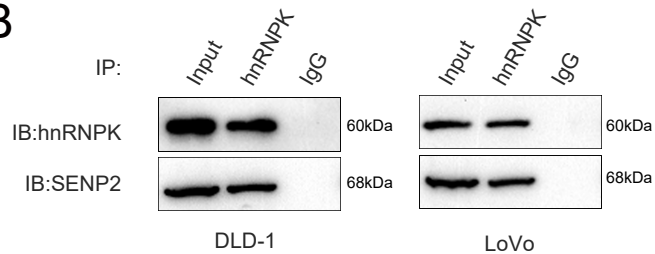**D**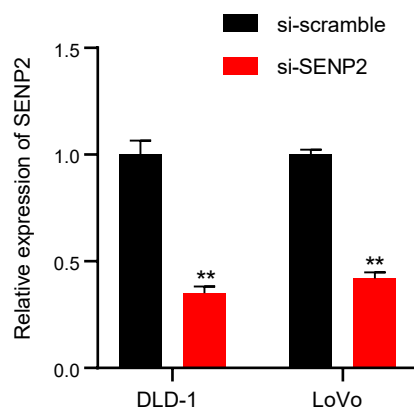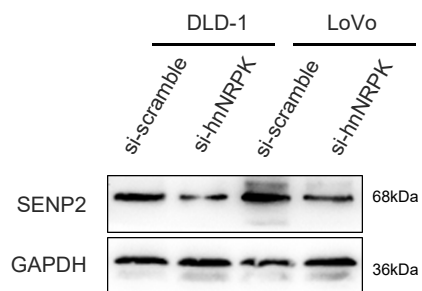**C**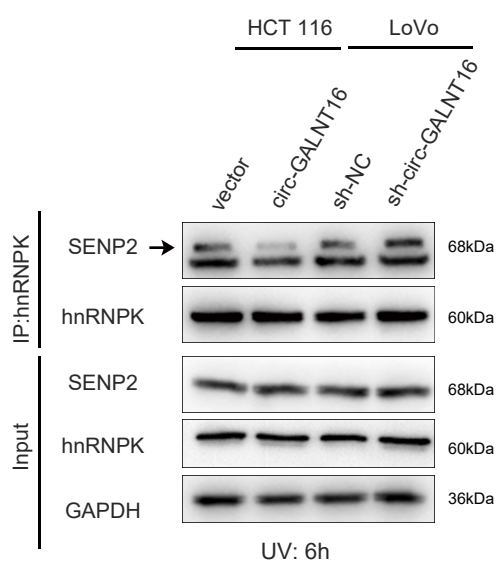**E**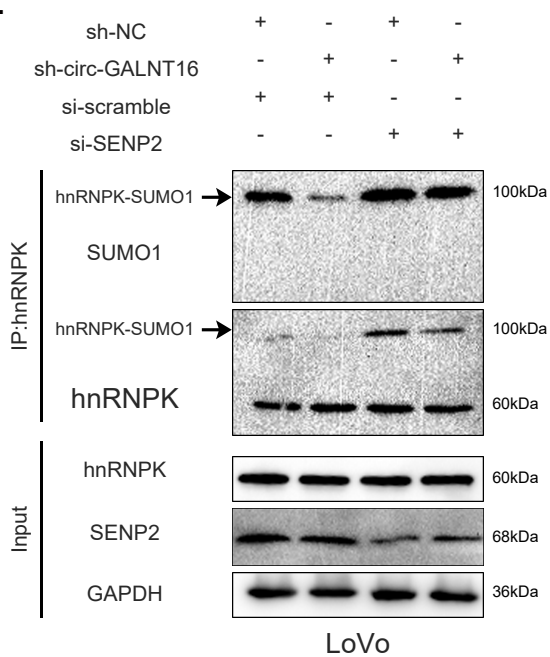**F**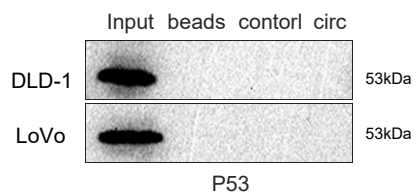**G**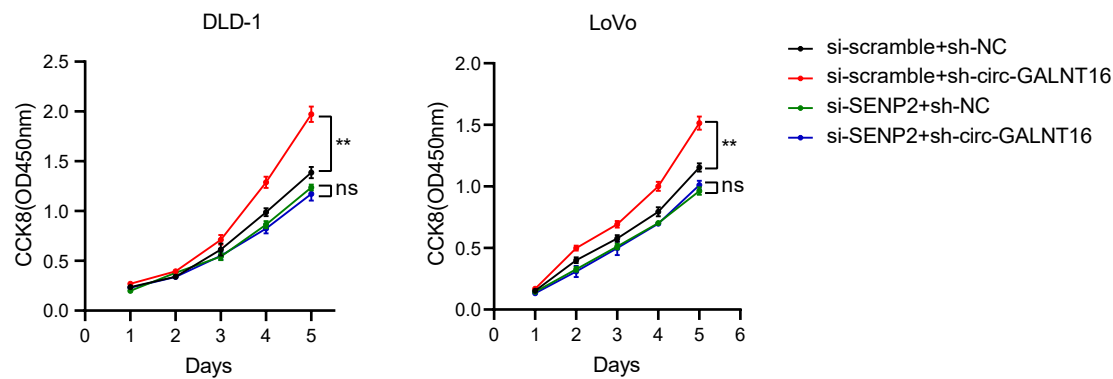

Supplement: Supplementary file 5 — Additional file 5: Figure S5. Circ-GALNT16 promotes the interaction between hnRNPK and p53 via inhibiting SENP2-mediated deSUMOylation. a. SUMOylation modification analysis was performed to identify the levels of hnRNPK SUMOylation in circ-GALNT16 knockdown and overexpression cells. b. The co-IP assay showed SENP2 could interact with hnRNPK in CRC cells. c. The co-IP assay was performed between SENP2 and hnRNPK in circ-GALNT16 silencing and overexpression cells at 6 h after UV stimulation. d. The knockdown efficiency of SENP2. e. SUMOylation modification analysis to explore the levels of hnRNPK SUMOylation in circ-GALNT16 knockdown and SENP2 knockdown cells. f. Pulldown assay showed p53 did not physically interact with circ-GALNT16. g. CCK8 indicated that circ-GALNT16 suppressed the proliferation of CRC by attenuating the deSUMOylation of hnRNPK. All data are presented as the means ± SD of three independent experiments.nsp>0.05, **p < 0.01. [file 13046_2021_2074_MOESM5_ESM.pdf]

**A**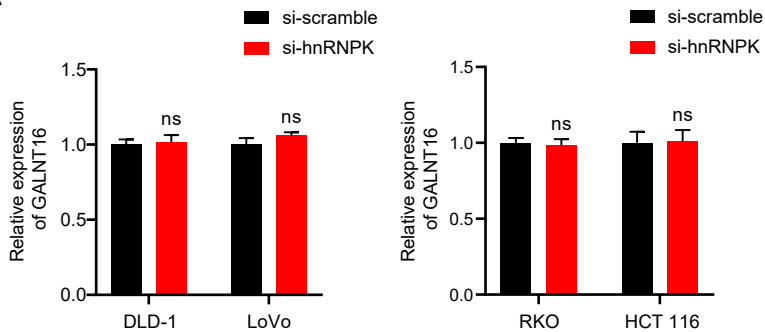**B**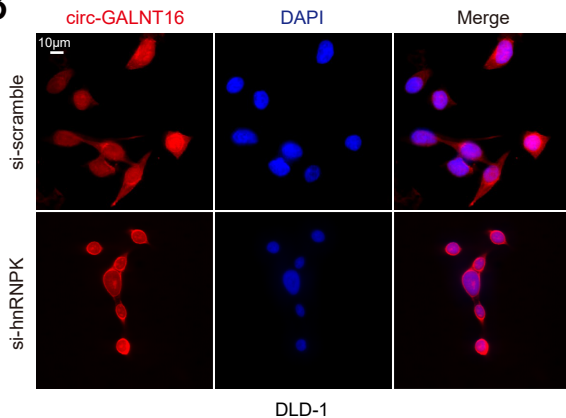**C**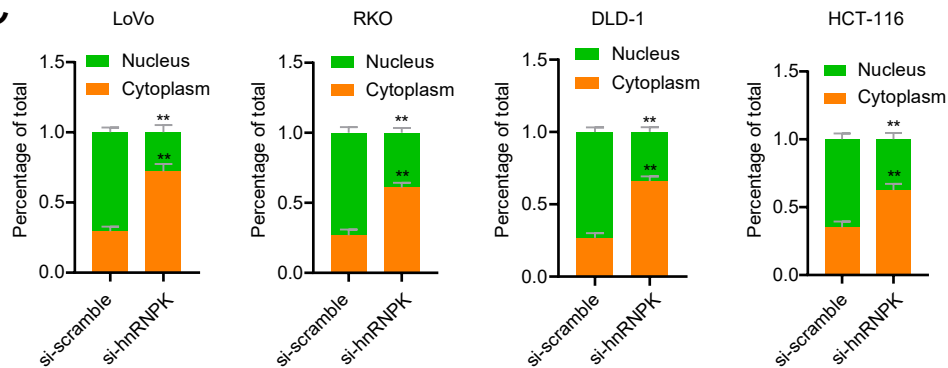

Supplement: Supplementary file 6 — Additional file 6: Figure S6. HnRNPK enhances the nuclear accumulation of circ-GALNT16. a. The mRNA expression level of GALNT16 in hnRNPK depletion cells. b-c. RNA FISH and subcellular fractionation assay again in hnRNPK-depletion cells. All data are shown as mean ± SD of three independent experiments. nsp >0.05, **p < 0.01. [file 13046_2021_2074_MOESM6_ESM.pdf]

**A**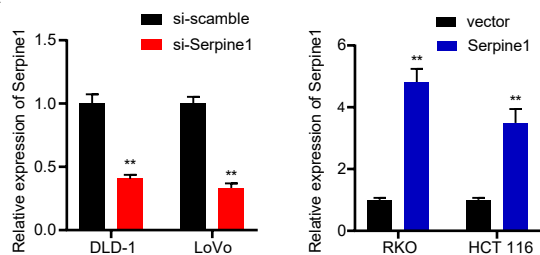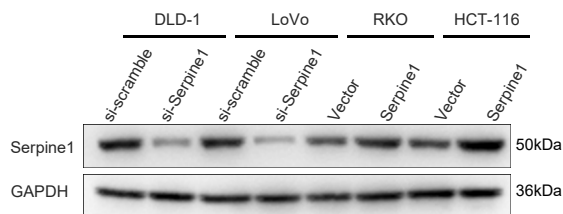**B**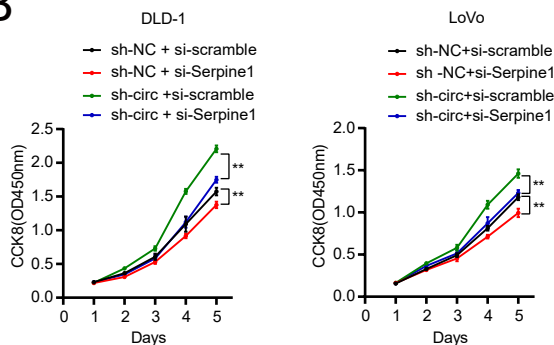**C**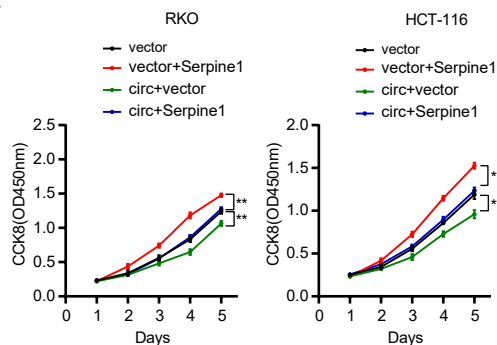**D**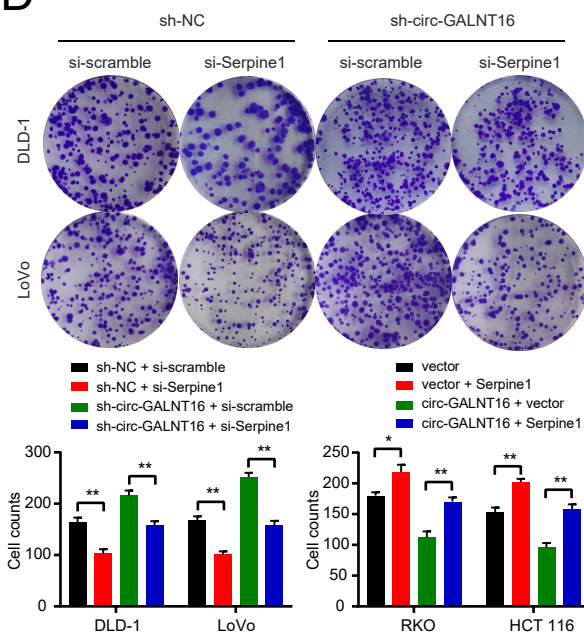**E**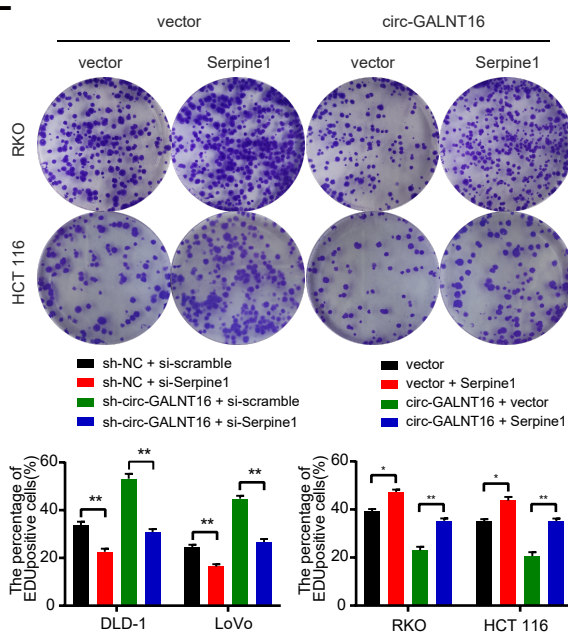**F**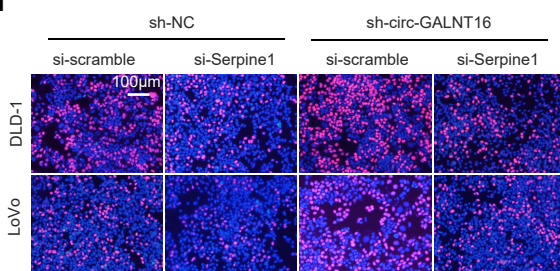**G**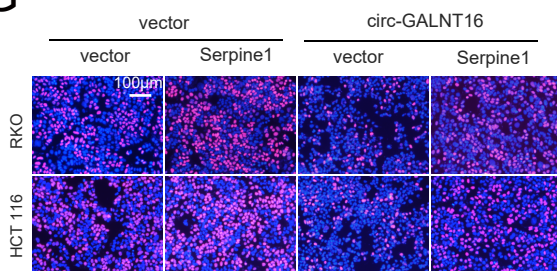

Supplement: Supplementary file 7 — Additional file 7: Figure S7. Circ-GALNT16 suppresses the proliferation ability of CRC cells through downregulating Serpine1. a. The knockdown and overexpression efficiency of Serpine1. b-g. CCK-8, colony formation, EdU assays were carried out in relatively treated cells targeted circ-GALNT16 and Serpine1. All data are shown as mean ± SD of three independent experiments. *p< 0.05, **p < 0.01. [file 13046_2021_2074_MOESM7_ESM.pdf]

A

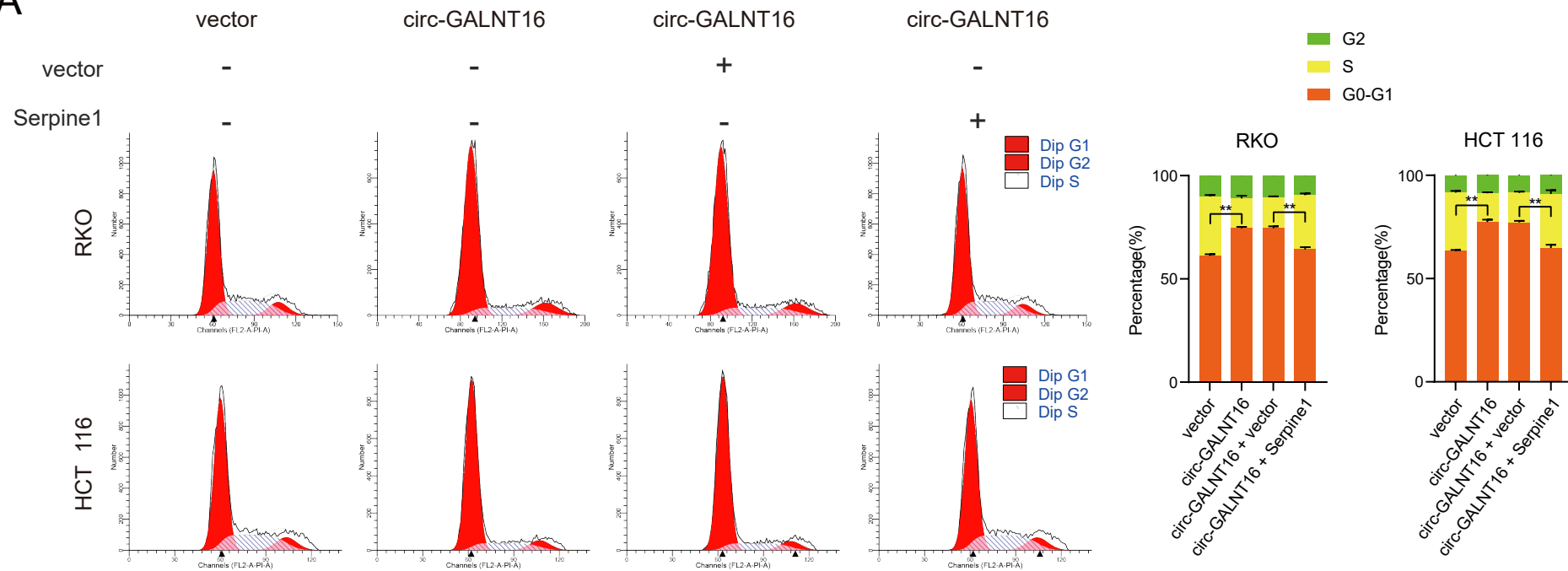

B

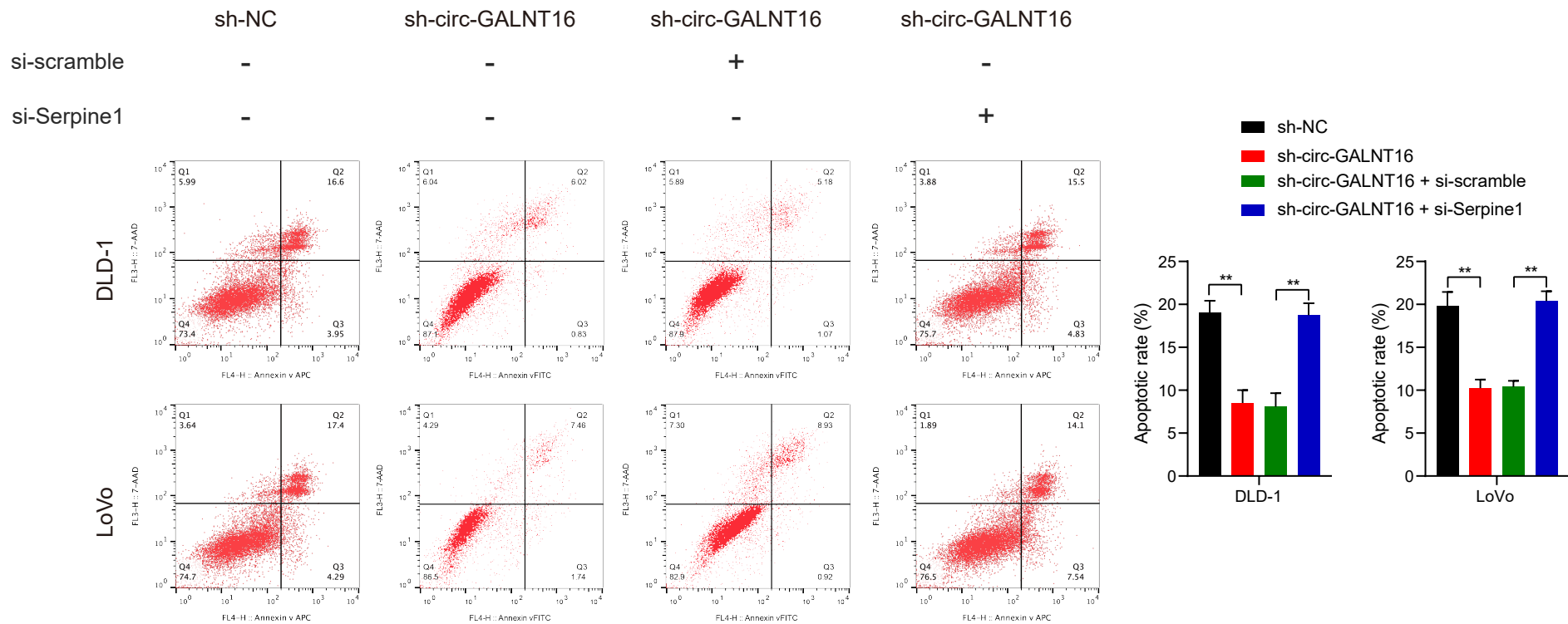

Supplement: Supplementary file 8 — Additional file 8: Figure S8. Circ-GALNT16 regulates cell cycle and apoptosis of CRC cells through downregulating Serpine1. a. Cell cycle assays were performed in circ-GALNT16 overexpression and Serpine1 overexpression groups. b. Cell apoptosis assays were performed in sh-circ-GALNT16 and si-Serpine1 co-transfected cells. **p < 0.01. [file 13046_2021_2074_MOESM8_ESM.pdf]

A

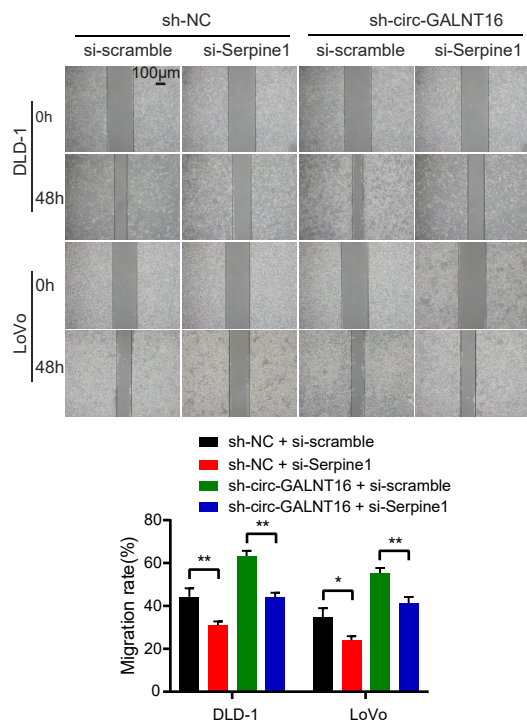

B

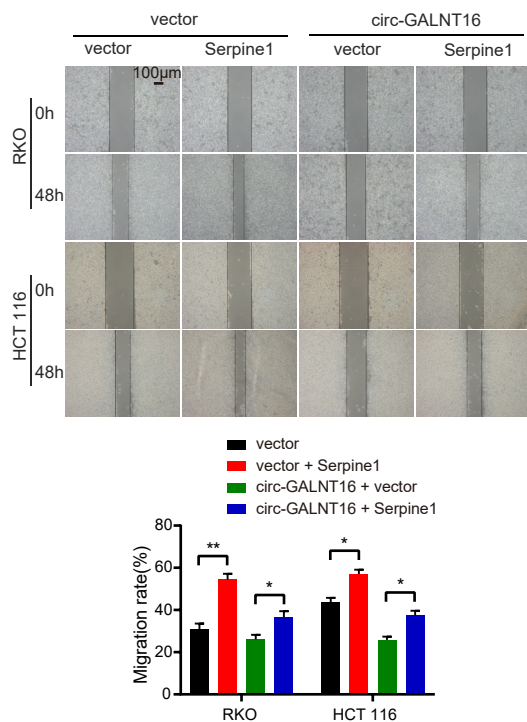

C

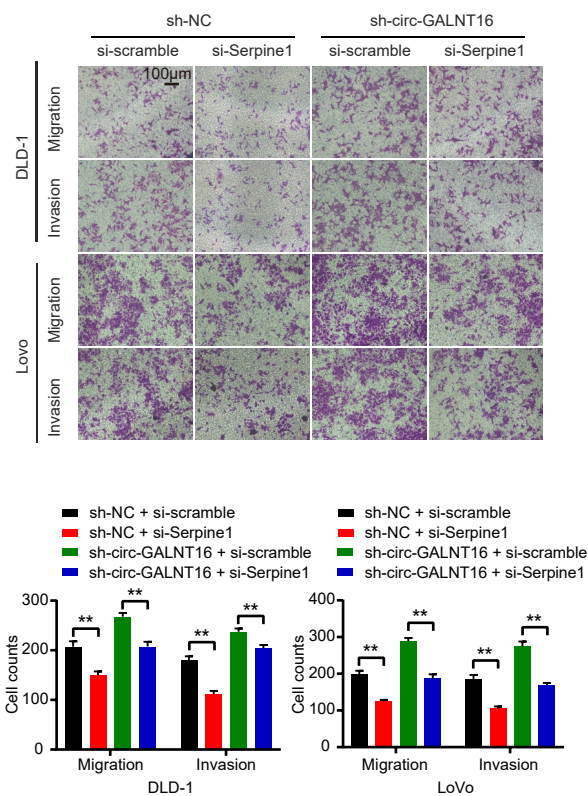

D

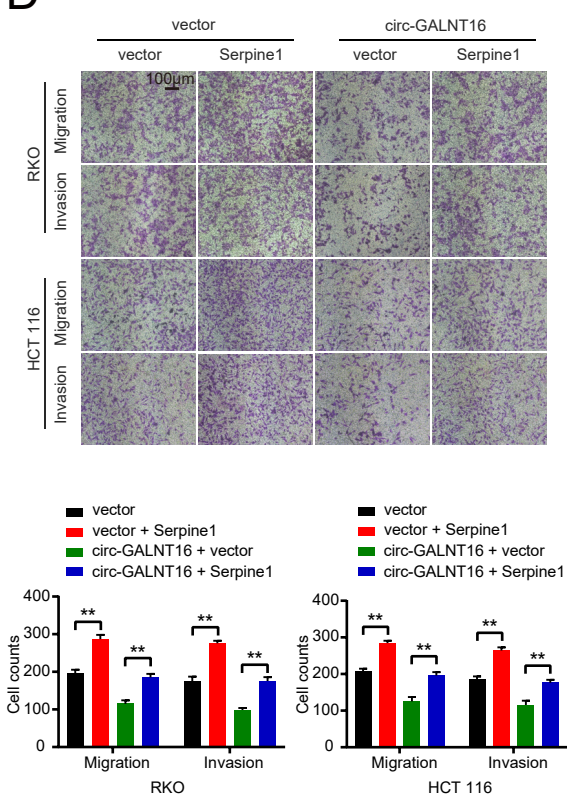

Supplement: Supplementary file 9 — Additional file 9: Figure S9. Circ-GALNT16 suppresses the metastasis ability of CRC cells through Serpine1. a-d. Transwell and wound healing assays were performed in relatively treated cells targeted circ-GALNT16 and Serpine1. All data are shown as mean ± SD of three independent experiments. *p < 0.05, **p < 0.01. [file 13046_2021_2074_MOESM9_ESM.pdf]

**A**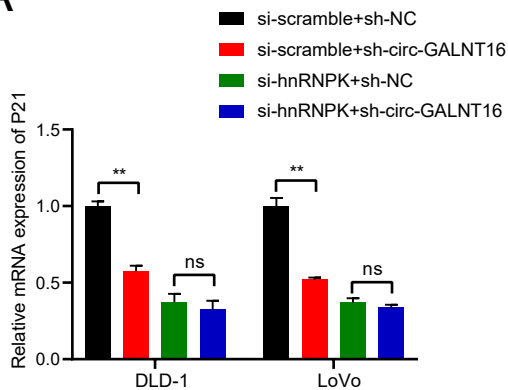**B**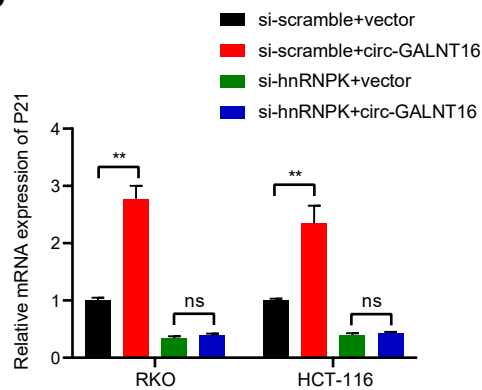**C**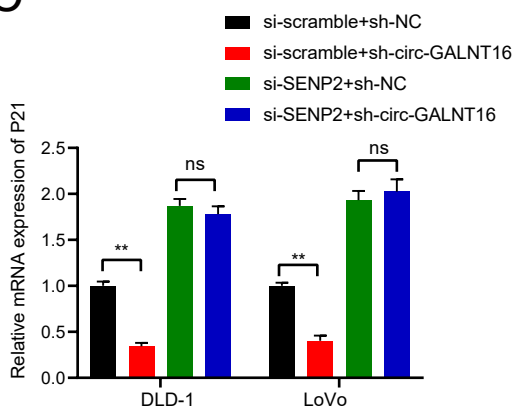**D**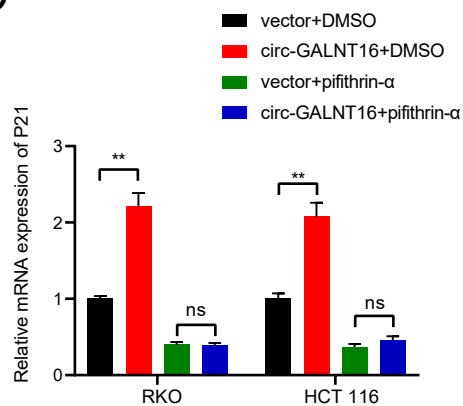**E**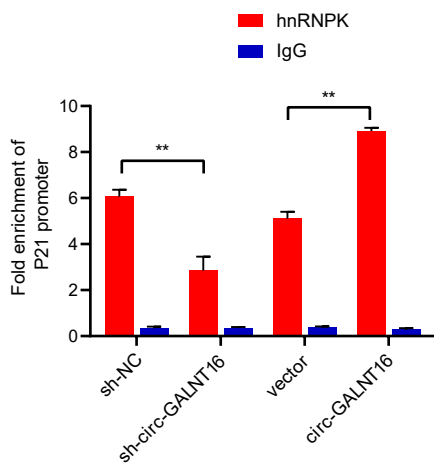**F**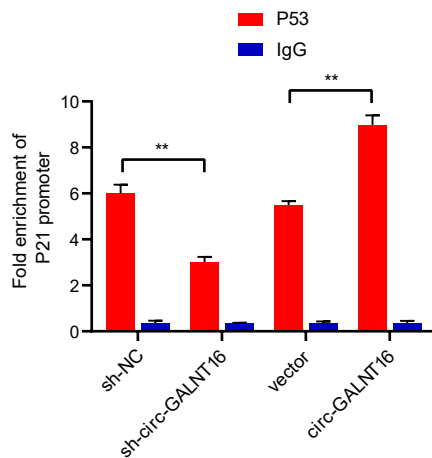

Supplement: Supplementary file 10 — Additional file 10: Figure S10. Circ-GALNT16 promotes p21 mRNA expression level through the SUMOylation of hnRNPK and p53. a, b. The mRNA level of p21 in circ-GALNT16 knockdown and overexpression cells while hnRNPK was silenced. c. The expression of p21 in circ-GALNT16 knockdown cells while SENP2 was silenced. d. The expression of p21 in circ-GALNT16 overexpression cells with the Pifithrin-α (10μM) treatment. e, f. HnRNPK and p53 chromatin immunoprecipitation were performed to measure the hnRNPK and p53 enrichment at the promoter region(s) of p21 in DLD-1 and RKO. All data are shown as mean ± SD of three independent experiments. nsp > 0.05, **p < 0.01. [file 13046_2021_2074_MOESM10_ESM.pdf]
