# Supplementary material for: Circ-GALNT16 restrains colorectal cancer progression by enhancing the SUMOylation of hnRNPK
Source: J Exp Clin Cancer Res. 2021 Aug 27;40:272. doi: 10.1186/s13046-021-02074-7 (PMC8400830; doi:10.1186/s13046-021-02074-7)
Supplement: Supplementary file 4 — Additional file 4: Figure S4. Circ-GALNT16 could specifically interact with hnRNPK in the nucleus and suppress the proliferation and metastasis through binding to hnRNPK. a. MS analysis results of hnRNPK. b. The knockdown efficiency of hnRNPK. c. Pulldown and silver staining performed in nucleoprotein of DLD-1 cells. d, e. CCK8 and transwell showed that circ-GALNT16 suppressed the proliferation and metastasis of CRC by interacting with hnRNPK. f. The mRNA expression level of hnRNPK in circ-GALNT16 knockdown and overexpression cells. All data are presented as the means ± SD of three independent experiments. nsp >0.05, *p < 0.05, **p < 0.01. [file 13046_2021_2074_MOESM4_ESM.pdf]

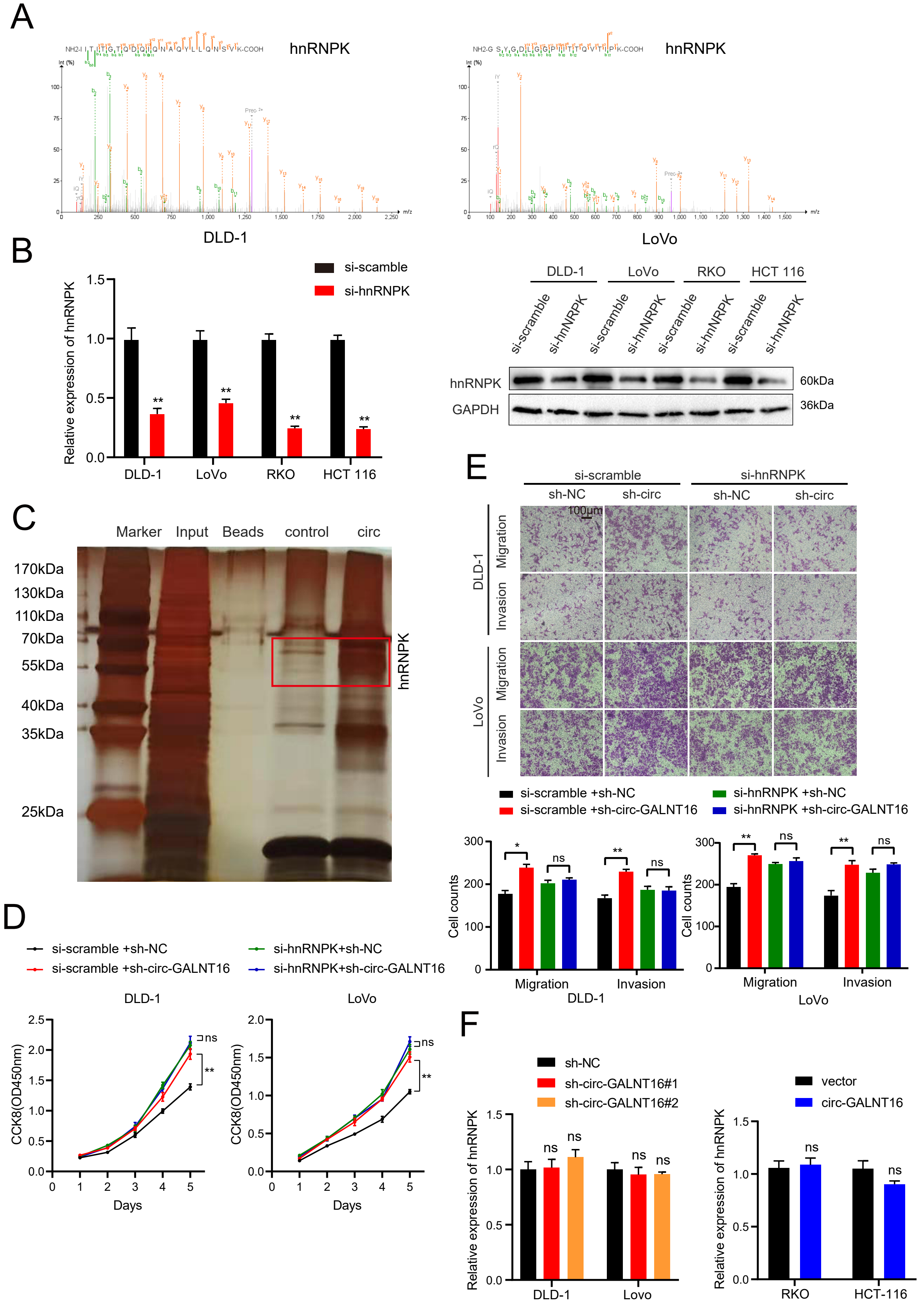
A
hnRNPK
DLD-1
hnRNPK
LoVo
B
si-scamble
si-hnRNPK
Relative expression of hnRNPK
DLD-1
LoVo
RKO
HCT 116
DLD-1
LoVo
RKO
HCT 116
si-scramble
si-hnRNPK
hnRNPK
GAPDH
60kDa
36kDa
C
Marker
Input
Beads
control
circ
170kDa
130kDa
110kDa
70kDa
55kDa
40kDa
35kDa
25kDa
hnRNPK
D
si-scramble +sh-NC
si-scramble +sh-circ-GALNT16
si-hnRNPK+sh-NC
si-hnRNPK+sh-circ-GALNT16
DLD-1
LoVo
CCK8(OD450nm)
Days
E
si-scramble
si-hnRNPK
sh-NC
sh-circ
100μm
DLD-1
LoVo
Migration
Invasion
si-scramble +sh-NC
si-scramble +sh-circ-GALNT16
si-hnRNPK +sh-NC
si-hnRNPK +sh-circ-GALNT16
Cell counts
Migration
Invasion
DLD-1
LoVo
F
sh-NC
sh-circ-GALNT16#1
sh-circ-GALNT16#2
Relative expression of hnRNPK
DLD-1
Lovo
vector
circ-GALNT16
RKO
HCT-116
