# Supplementary material for: Circ-GALNT16 restrains colorectal cancer progression by enhancing the SUMOylation of hnRNPK
Source: J Exp Clin Cancer Res. 2021 Aug 27;40:272. doi: 10.1186/s13046-021-02074-7 (PMC8400830; doi:10.1186/s13046-021-02074-7)
Supplement: Supplementary file 14 — Additional file 14: Table S4. Primary antibodies information used in this study. [file 13046_2021_2074_MOESM14_ESM.docx]

| **Table. S4** | | |
| --- | --- | --- |
| **Antigens** | **Manufacturer** | **Application** |
| hnRNPK | Proteintech: 11426 | 1:500 for WB; 4.0 ug for IP |
| Flag | Abcam: ab205606 | 1:500 for WB; 1:30 for IP |
| p53 | Cell Signaling Technology:2524 | 1:1000 for WB; 1:100 for IP |
| SUMO1 | Abcam: ab32058 | 1:1000 for WB |
| SENP2 | Abcam: ab131637 | 1:2000 for WB |
| Serpine1 | Proteintech: 66261 | 1:5000 for WB; 1:50 for IHC |
| Ki-67 | Abcam: ab15580 | 1:500 for IHC |
| C-myc | Abcam: ab32072 | 1:200 for IHC |
| AKT | Abcam: ab8805 | 1:500 for WB |
| P-AKT | Abcam: ab38449 | 1:500 for WB |
| Cyclin D1 | Abcam: ab134175 | 1:5000 for WB |
| CDK4 | Abcam: ab108357 | 1:1000 for WB |
| Bcl-2 | Abcam: ab32124 | 1:1000 for WB |
| Bax | Abcam: ab32503 | 1:1000 for WB |
| GAPDH | Abcam: ab9485 | 1:2500 for WB |
